# Genus-wide comparative genomics of *Colletotrichum* reveals evolutionary conservation and divergent ecological adaptations

**DOI:** 10.3897/imafungus.17.195303

**Published:** 2026-08-17

**Authors:** Jian-Xin Shen, Min Qiao, Jia-Nan Hong, Xing-Wen Dai, Wen-Jie Li, Shu-Qun Liu, Ze-Fen Yu

**Affiliations:** 1 State Key Laboratory for Conservation and Utilization of Bio-Resources in Yunnan, Yunnan University, Kunming 650504, China School of Life Sciences, Yunnan University Kunming China; 2 Yunnan Key Laboratory of Basic Research and Innovative Application for Green Biological Production, Yunnan University, Kunming 650504, China State Key Laboratory for Conservation and Utilization of Bio-Resources in Yunnan, Yunnan University Kunming China; 3 School of Life Sciences, Yunnan University, Kunming 650504, China Yunnan Key Laboratory of Basic Research and Innovative Application for Green Biological Production, Yunnan University Kunming China

**Keywords:** Adaption, comparative genomics, evolution, pathogenic fungi

## Abstract

*Colletotrichum* spp. are widespread fungal pathogens that cause anthracnose in numerous economically important crops and, exhibiting extensive taxonomic, host plant, and lifestyle diversity. Here, we analyzed the genome sequences of 150 strains representing 97 species across 15 species complexes and four singletons, including and integrating both newly assembled and publicly available genomes. Phylogenomic investigation clarified the taxonomy of *Colletotrichum* and resolved misidentifications. We identified variations in genome architecture contributed by phylogenetic lineages, host types, and lifestyles, with transposable element proliferation playing significant roles. Interestingly, codon usage bias followed phylogenetic patterns, with species complexes forming distinct clusters and exhibiting a significant bistable co-evolutionary relationship with tRNA genes. Functional gene repertoires displayed coordinated shifts, with higher abundance in broad host-range species complexes and in strains associated with woody or dicotyledonous hosts. Although most functional categories retained strong phylogenetic signals, co-occurrence analysis of weak-signal categories identified modules related to host cell wall disruption, fungal cell wall remodeling, and virulence that were significantly associated with ecological differentiation. Evolutionary trajectories and gene family dynamics further revealed divergent ecological strategies, with oxidative versus rapid-response detoxification in woody- and herbaceous-associated lineages, respectively. The diversifications were accompanied by woody-specific expansion of GH39 and alkaline proteases and progressive differentiation of pectin-degrading capacity, including contraction in woody lineages and divergence between dicot- and monocot-associated herbaceous lineages. The *C. gloeosporioides* species complex emerged with a comprehensive expansion of detoxification and cell wall-degrading capacities, likely contributing to its broad host range. In contrast, endophytic lineages exhibited convergent gene family contraction in adhesion and cell wall remodeling. Together, this study revealed concordance of codon usage and functional gene abundance with phylogeny, along with diverse host- and lifestyle-associated adaptive strategies in this important group of plant pathogens.

## Introduction

*Colletotrichum*, the sole genus in the family *Glomerellaceae* within the order *Glomerellales (Sordariomycetes)*, is widely distributed and recognized as one of the most ten significant plant pathogens (Dean et al. 2012). Currently, *Colletotrichum* includes 1,104 entries in Index Fungorum (http://indexfungorum.org/; Accessed April 2026), with 344 species grouped into 20 species complexes (Liu et al. 2022; Talhinhas and Baroncelli 2023). It infects over 760 host species, primarily monocots like *Poaceae* and *Orchidaceae*, and dicots such as *Fabaceae*, *Rosaceae*, and *Solanaceae*, displaying different taxonomic levels of host specificity (species, genus, or family) (Talhinhas and Baroncelli 2021, 2023). In addition, *Colletotrichum* spp. also cause disease of animals and humans (Manire et al. 2002; Hung et al. 2020; Jayawardena et al. 2021; Talhinhas and Baroncelli 2023).

A hemi-biotrophic infection strategy predominates in *Colletotrichum*, and diversification across species complexes is commonly associated with host shifts. The *C. gloeosporioides* species complex (Glosc) represents the lineage with the broadest host range, distributed across angiosperms, whereas the *C. acutatum* species complex (Acusc), although also exhibiting a broad host range, mainly infects dicot woody fruit trees and rarely occurs on monocots (Jayawardena et al. 2021; Talhinhas and Baroncelli 2023). In contrast, species complexes with narrower host ranges are predominantly associated with herbaceous plants (dicot and monocot). The *C. graminicola* (Grasc) and *C. caudatum* (Causc) species complexes are typically regarded as specialized on monocot herbaceous hosts (Crouch et al. 2009b; Crouch 2014). Broad host ranges are often linked to the ability to infect woody hosts.

Distinct from the clear phylogenetic associations with host types, *Colletotrichum* displays a continuum of lifestyles, with pathogenic and endophytic species interspersed within lineages (Hiruma 2020). Many species function as both pathogens and endophytes, and even minor genetic or regulatory variation can drive mutualist-pathogen transitions (Hiruma et al. 2023; Newfeld et al. 2025; Ujimatsu et al. 2025). However, in the Gigasporium (Gigsc) and tibetense (Tibsc) species complexes, some endophytic species cluster into distinct lineages, suggesting more stable lifestyle differentiation (Liu et al. 2022; Zheng et al. 2022). These diverse and interwoven ecological contexts complicate our understanding of *Colletotrichum* evolution and adaptation.

Genomic analyses have provided important insights into the genetic basis of ecological variation in *Colletotrichum*. Despite phylogenetic divergence, the broad host-range of Glosc and Acusc show convergent expansion of carbohydrate-active enzyme (CAZyme) and protease repertoires, whereas the monocot-specialized Grasc exhibits reduced plant cell wall-degrading enzymes, especially pectinases (Baroncelli et al. 2016; Zhang et al. 2023a; Baroncelli et al. 2024). Moreover, repeat-mediated chromosomal rearrangements have been shown to play a key role in shaping host range differentiation (Ma et al. 2025b). The monocot-adapted Grasc also exhibits contraction of detoxification-related gene families such as RTA1 and cytochrome P450 monooxygenases, whereas Acusc shows expansion of necrosis- and ethylene-inducing peptide 1-like proteins (NLPs), reflecting adaptation to dicot hosts (Baroncelli et al. 2016; Liang et al. 2018; Ortiz-Álvarez et al. 2024). In *C. gloeosporioides*, adaptation to woody hosts is associated with expansion of plant surface signal recognition genes, particularly GH17 and the MP65, compared to those infecting herbaceous-associated strains (Meng and Tian 2023).

Genomic evidence has also revealed differences among *Colletotrichum* lifestyles. Comparative analyses of pathogenic and endophytic *C. gloeosporioides* strains indicate that endophytes have lost pathogenicity-related genes or regions (e.g., *CgDN3*, *Cap20*, and polyketide synthase clusters), reduced pectin-degrading enzymes, and enriched carbon acquisition-related function (Fu et al. 2020; Yue et al. 2025). A similar pattern is observed in *C. fructicola*, where endophytic strains exhibit loss of pathogenic lineage-specific regions associated with secondary metabolism (Liang et al. 2024). In *C. scovillei*, the endophytic state is characterized by the loss of a 34-kb genomic fragment and concomitant reductions in effector and transposable elements (Hsieh et al. 2022). However, current studies are largely confined to comparisons within similar genetic backgrounds, leaving the shared features of *Colletotrichum* across comparable ecological contexts poorly characterized.

Codon usage bias (CUB), the non-random use of synonymous codons, is a pervasive genomic feature linked to gene expression and functional adaptation. In fungi, this bias is predominantly shaped by adaptive selection and coordinated with tRNA pools to enhance translational efficiency (Wint et al. 2022). Comparative analyses indicate that codon optimization is associated with host range, with higher levels observed in generalist than specialist fungal pathogens (Badet et al. 2017). Meanwhile, host plant type may also shape codon usage, as fungal pathogens often exhibit patterns resembling those of their hosts (Gupta and Singh 2021; Nisa et al. 2024). Beyond host-related factors, CUB has also been linked to lifestyle transitions. For example, Guerreiro et al. (2026) showed that adaptive translation facilitates the shift from saprotrophy to opportunistic human pathogenicity in the order *Trichosporonales*, particularly through codon optimization of metabolic genes, rather than changes in overall genome composition. Similarly, analyses of single-copy core genes in *Fusarium* revealed significant lifestyle-associated differences in codon optimization, with markedly lower levels observed in insect mutualists compared with endophytes, plant pathogens, and saprotrophs (Hill et al. 2022). CUB contributes to multiple aspects of ecological adaptation in fungi. Although codon usage in *Colletotrichum* has been investigated in mitochondrial genomes (Wu et al. 2025), genome-wide analyses remained largely unexplored.

Functional studies in *Colletotrichum* have identified numerous genes essential for host infection, including those encoding secreted proteins (e.g., CAZymes, proteases, and effectors), transporters, and transcription factors. Among CAZymes, pectate lyases, cutinases, β-1,3-glucanases, laccases, and lytic polysaccharide monooxygenases (AA9) are important for *Colletotrichum* morphogenesis, cuticle penetration, appressorium-mediated invasion, and suppression of host defense responses (Yakoby et al. 2001; Auyong et al. 2015; Wei et al. 2017; Gu et al. 2024; Ma et al. 2025a). An extracellular serine protease (sp78) in *C. coccodes* is required for virulence, whereas a metalloprotease (Cgfl) in *C. graminicola* contributes to immune evasion by degrading host chitinases (Redman and Rodriguez 2002; Sanz-Martín et al. 2016). Diverse effector proteins, such as Vne1, CfEC92, LysM effectors, SIB1, and NIS1, suppress host immunity and are necessary for full pathogenicity (Takahara et al. 2016; Irieda et al. 2019; Shang et al. 2020; Zhang et al. 2021; Wang et al. 2026).

Beyond mediating nutrient uptake and detoxification, transporters—including ATP-binding cassette (ABC), major facilitator superfamily (MFS), as well as copper and ammonium transporters—also broadly support fungal development and pathogenicity (Barhoom et al. 2008; Shnaiderman et al. 2013; Chen et al. 2019; Oliveira et al. 2022). Transcription factors function throughout the *Colletotrichum* infection cycle, with C*_2_*H_2_ zinc finger (SltA/CrzA/Con7), homeobox (CoHox3/CgHox7), Zn_2_Cys_6_ (CLTA1), and bZIP (CsBzip10) regulators playing key roles in sporulation and germination, appressorium formation, and biotrophic–necrotrophic transition (Dufresne et al. 2000; Dubey et al. 2016; Yokoyama et al. 2018; Liu and Li 2019; Huang et al. 2024; Zhou et al. 2024). Additionally, *Colletotrichum* produce diverse secondary metabolites that contribute to disease development, host modulation, and ecological competition (Moraga et al. 2018). Although Chen et al. (2022) systematically analyzed the composition of the secretome and secondary metabolite gene clusters (SMBGCs) across many *Colletotrichum* strains and compared their variation among species complexes, comparable analyses of transporters and transcription factors are lacking, and how these functional gene repertoires vary across ecological niches remained unclear.

To date, more than 300 *Colletotrichum* genomes are available, spanning diverse species, host origins, and lifestyles. However, integrative analyses across these diverse datasets have yet to be conducted. Here, we combine newly assembled and publicly available genomes to perform a genus-wide comparative genomic analysis across multiple dimensions, including species complexes, lifestyles, host life-form and clade. This study aims to: 1) establish a genome-scale phylogenomic framework and estimate divergence times across *Colletotrichum*; 2) characterize genome structural features, their relationship, underlying mechanisms, and variation across phylogenetic and ecological contexts; 3) investigate codon usage bias patterns and their co-evolution with tRNA; 4) analyze the composition and variation of functional gene repertoires, and identify functional categories associated with ecological differentiation; 5) infer adaptive strategies underlying ecological diversification.

## Materials and methods

### Genome collection, grouping and assessment

The 20 species of *Colletotrichum* sequenced in this study were preserved at the Microbial Library of the Germplasm Bank of Wild Species from Southwest China (Suppl. material 2: table S1). Strains were cultured on potato dextrose agar medium at 28 °C for 7–14 days and subsequently transported on dry ice to the Institute of Microbiology, Chinese Academy of Sciences, for *de novo* sequencing. Raw paired-end sequencing data were quality assessed using FastQC v0.11.9 (Andrews 2017). Genome assembly was performed using spades v3.15.5 (Prjibelski et al. 2020) with the parameters “--careful -k 21,33,55,77,99” for optimal accuracy. Alternatively, existing *Colletotrichum* genome data were retrieved from National Center for Biotechnology Information (NCBI; https://www.ncbi.nlm.nih.gov/; Accessed 1^st^ April 2024). Strains were grouped according to species complexes, lifestyles, the clade and life-forms of host plant from where they were isolated. Filtering was applied considering grouping requirements and different genome size, resulting in a final genomic dataset comprising 130 strains from 81 species. Most genome assemblies included in this study have been previously published, and the corresponding accession numbers and original references are provided in Suppl. material 2: table S1. Type strain genomes were included whenever available to ensure taxonomic representativeness, and genomes lacking associated publications (e.g., direct submissions) are explicitly indicated.

To reduce assembly artifacts and ensure data consistency, contigs shorter than 1,000 bp were excluded. This threshold is widely used in fungal comparative genomics. Genome contiguity and completeness were then assessed using QUAST v5.2.0 (Gurevich et al. 2013) and BUSCO v5.5.0 (Manni et al. 2021), respectively. The “sordariomycetes_odb10” serves as the benchmarking dataset in BUSCO.

### Genome annotation

RepeatMasker v4.1.6 (Smit et al. 2015) with the repeat libraries Repbase Edition-20181026 (Jurka et al. 2005) and Dfam release 3.8 (Storer et al. 2021), in combination with RepeatModeler v2.0.5 (Flynn et al. 2020), were used to identify repetitive sequences. Transfer RNA (tRNA) genes were predicted using tRNAscan-SE v2.0.12 (Chan et al. 2021). Gene annotation was preformed using the comprehensive genome annotation pipeline Maker v3.1.4 (Campbell et al. 2014), which integrated homology-based gene prediction from tBLASTx (Camacho et al. 2009) and *ab initio* gene prediction from SNAP (Korf 2004). Initial homology-based prediction followed by two successive rounds of *ab initio* prediction were conducted according to standard procedures. For improved accuracy, the homology prediction step was guided by CDS and protein sequences derived from 2–3 closely related strains or species (Suppl. material 3: table S2). Intergenic distances were defined as the shorter distance between the 5’- and 3’-ends of adjacent genes, calculated using in-house scripts (https://github.com/JianxinS0806-Git/2026_IMA_scripts).

### Codon analysis

To ensure accuracy, predicted coding sequences (CDS) were refined by removing those without start codons, correcting those with multiple stop codons, and requiring a minimum length of 100 bp. Subsequently, in-house python scripts (https://github.com/JianxinS0806-Git/2026_IMA_scripts) were employed to calculate the frequency of base at the third codon position, GC1s, GC2s, GC3s, and relative synonymous codon usage (RSCU) for each strain and its CDS. The RSCU formula is as follows:

${\mathrm{RSCU}}_{ij}=\frac{X_{ij}}{\frac{1}{n_{j}}\sum_{k=1}^{n_{j}}X_{kj}}$ (1)

*RSCU_ij_* represents the RSCU value for the i-th codon of the j-th amino acid. *X_ij_* denotes the occurrence count of the i-th codon in the CDS or strains. *n_ij_* is the total number of synonymous codons for amino acid j. A codon with an RSCU value of 1 is used at it expected frequency, while deviations indicate usage bias. Accordingly, codons were classified into three categories: high bias (RSCU > 1.5), no bias (0.5 ≤ RSCU ≤ 1.5), and low bias (RSCU < 0.5).

CodonW v1.4.2 (John 1999) was used to calculate the effective number of codons (ENc) for each CDS was calculated. The calculation is detailed in formula 2.

$ENc=2+\frac{N-1}{\sum_{i=1}^{k}f_{i}}$ (2)

Where *N* represents the total number of codons, while *k* is the count of distinct codon types within the CDS. *f_i_* denotes the frequency of the i-th codon. The ENc value ranges from 20 to 61, reflecting codon usage diversity. Lower ENc values indicate more concentrated codon usage, whereas higher values signify more uniform usage.

### Functional annotation

InterProScan v5.68–100 (Jones et al. 2014) was utilized alongside Pfam database v37.0 (Mistry et al. 2021) to classify proteins into appropriate families. The major effector and transcription factor families were identified based on their Pfam accession numbers (Suppl. material 3: table S3). Diamond v2.1.9 (Buchfink et al. 2021) was employed to annotate the complete protein-coding genes coding for proteases, and transporters that were drawn from multiple resources, including the UniProtKB fungal dataset (https://ftp.uniprot.org/pub/databases/uniprot/current_release/knowledgebase/taxonomic_divisions/; Accessed 1^st^ July 2024) (UniProt Consortium 2023), MEROPS (https://www.ebi.ac.uk/merops/; Accessed 11^th^ August 2024) (Rawlings et al. 2018), Transporter Classification Database (TCDB; https://www.tcdb.org/; Accessed 10^th^ September 2024) (Saier et al. 2021). SignalP v6.0 (Teufel et al. 2022) and TMHMM v2.0 (Krogh et al. 2001) were combined to predict signal peptides and transmembrane helices respectively, for identifying fungal secretomes. dbCAN3 (Zheng et al. 2023) and EffectorP v3.0 (Sperschneider and Dodds 2022) facilitated the identification of CAZymes and effectors, respectively.

AntiSMASH v7.1.0 (Blin et al. 2023) was preformed to annotate and analyze secondary metabolite biosynthetic gene clusters (SMBCs). To improve the identification of SMBCs, the parameters “--fullhmmer” and “--clusterhmmer” were configured for comprehensive HMMER searches and clustering analyses; while “--cb-general”, “--cb-subclusters”, and “--cb-knownclusters” were used for comparison with known gene clusters. Major functional categories were selected based on prevalence and variability across genomes. For functional groups with large category sizes (CAZymes, proteases, transporters, and transcription factors), categories present in ≥30% of strains (n = 150) were retained, and those within the top 25%, 25%, 5%, and 25% of variance were selected, respectively. For groups with fewer categories, categories with consistently low abundance (typically 0–3 copies) and minimal variation across genomes were excluded, retaining four effector-related categories and six SMBGCs classes for subsequent analyses.

### Gene family analysis

The strain *Verticillium dahliae* VdLs. 17 (from the NCBI WGS project ABJE01; Accessed 7^th^ June 2024) was included as an outgroup in the analysis dataset, and gene families were inferred using OrthoFinder v2.5.5 (Emms and Kelly 2019) with default paraments. CAFE v5.0 (Mendes et al. 2021) was employed to assess the evolution and variation of gene families, encompassing expansions and contractions. To maintain the stability and validity of the model, gene families with the greatest disparity between maximum and minimum counts (≥ 8) among species were removed. A Poisson distribution model was selected for the root frequency distribution, with the number of rate categories for gene family variation set to 2, and the significance threshold for changes in family size was established at a *p*-value of less than 0.05.

### Phylogenomic reconstruction

The 160 single-copy orthologous genes identified by OrthoFinder v2.5.5 (Emms and Kelly 2019) were utilized for maximum-likelihood (ML) analysis, with amino acid sequences chosen over nucleotides for their greater robustness to synonymous substitutions and sufficient resolution when concatenated (65,708 site). Multiple sequence alignments were conducted with MAFFT v7.505 (Katoh and Standley 2013) using the “--auto” option, and all aligned protein sequence were concatenated by an in-house python script (https://github.com/JianxinS0806-Git/2026_IMA_scripts). Low-quality alignment regions were automatically trimmed using TrimAL v1.4.rev22 (Capella-Gutiérrez et al. 2009) with the “-automated1” option to improve accuracy. Finally, ML trees were reconstructed in RAxML v8.2.12 (Stamatakis 2014) base on the JTT substitution and Γ distribution, with 1000 rapid bootstrap replicates for robustness.

Meanwhile, additional type strains from a broader range of species were included, and a concatenated multilocus phylogeny was constructed using five commonly used genes: ITS, *gapdh*, *chs-1*, *act*, and *tub2*. For sequenced strains, gene fragments were downloaded from NCBI (Suppl. material 3: table S4), whereas for unsequenced loci, sequences were extracted from genome assemblies using homologous genes from type strains or species as queries. BLASTn v2.13.0 (Camacho et al. 2009) was used to identify genomic coordinates, and corresponding sequences were retrieved using SAMtools v1.6 (Danecek et al. 2021), with reverse-complement sequences corrected using Seqkit v2.13.0 (Shen et al. 2024). *Colletotrichum truncatum* KLC.C-4 was excluded from the multilocus alignment due to insufficient loci recovery (3/5 genes). Each locus was aligned using MAFFT v7.505 (Katoh and Standley 2013) with the “--auto” option, followed by manual inspection and trimming in MEGA v12.1 (Stecher et al. 2026). The five alignments were concatenated using FASconCAT v1.06 (Kück and Longo 2014), yielding a final alignment of 2,408 positions (*act*: 1–321, *chs-1*: 322–620, *gadph*: 621–1,014, ITS: 1,015–1,622, *tub2*: 1,623–2,408). A ML phylogeny was inferred using IQ-TREE v3.1.2 (Wong et al. 2026) with the “-m MFP+MERGE” option for partition-specific model selection and 1,000 rapid bootstrap replicates for branch support.

### Divergence time estimation

The penalized likelihood method (Sanderson 2002) was implemented in r8s v1.81 (Sanderson 2003), utilizing the truncated Newton algorithm to optimize the likelihood function for estimating divergence times in the ML tree. The fossil record of *Colletotrichum* is extremely limited, with the only available fossil (*Protocolletotrichum deccanensis*) providing minimal information for calibrating the crown node (Samarakoon et al. 2019). Moreover, older nodes typically involve greater temporal uncertainty and are less suitable for constraining internal divergence events. To better stabilize node age estimates across the tree, we therefore applied secondary calibrations using six nodes from TimeTree (https://timetree.org/; Accessed 12^th^ October 2024) (Kumar et al. 2022). The root node was set to the *Glomerellales* divergence (110 MYA), with additional calibrations for the divergence of the *C. spaethianum* species complex (Spasc; 7 MYA), the Grasc (6 MYA), the Acusc (5 MYA), the *C. orbiculare* species complex (Orbsc; 4 MYA), and the *C. destructivum* species complex (Dessc; 3 MYA).

### Statistics and visualization

Phylogenetic and time tree visualizations were generated via the iTOL server (https://itol.embl.de/) (Letunic and Bork 2024). Phylogenetic signal was assessed in R v4.5.2 using the “vegan v2.7.2” (Mantel test) and “phytools v2.5-2” (Blomberg’s K and Pagel’s λ) packages (Revell 2024; Oksanen et al. 2025; R Core Team 2025). Correlation networks of functional gene categories were constructed based on pairwise Spearman’s rank correlations, and visualized in Cytoscape v3.10.4, with module identified using the Leiden algorithm (Shannon et al. 2003; Zar 2014; Traag et al. 2019). Additional statistical analyses and data visualization were primarily performed using Python v3.9. The Kruskal-Wallis test (scipy v1.16.1 package; “scipy.stats.ruskal”) (Virtanen et al. 2020) and Dunn’s post-hoc test (scikit-posthocs v0.11.4 package; “scikit_posthocs.posthoc_dunn”) (Terpilowski 2019) were applied to examine differences among phylogeny/host clade (members ≥ 3), while the Mann-Whitney U test (scipy v1.16.1 package; “scipy.stats.mannwhitneyu”) examined differences between lifestyle/host life-form groups (members = 2). Linear regression analysis was performed with the “scipy.stats.linregress” function and visualization was generated using the “matplotlib v3.10.5” and “seaborn v0.13.2” packages (Hunter 2007; Waskom 2021).

## Results

### Phylogenetic analysis

**Figure 1.**
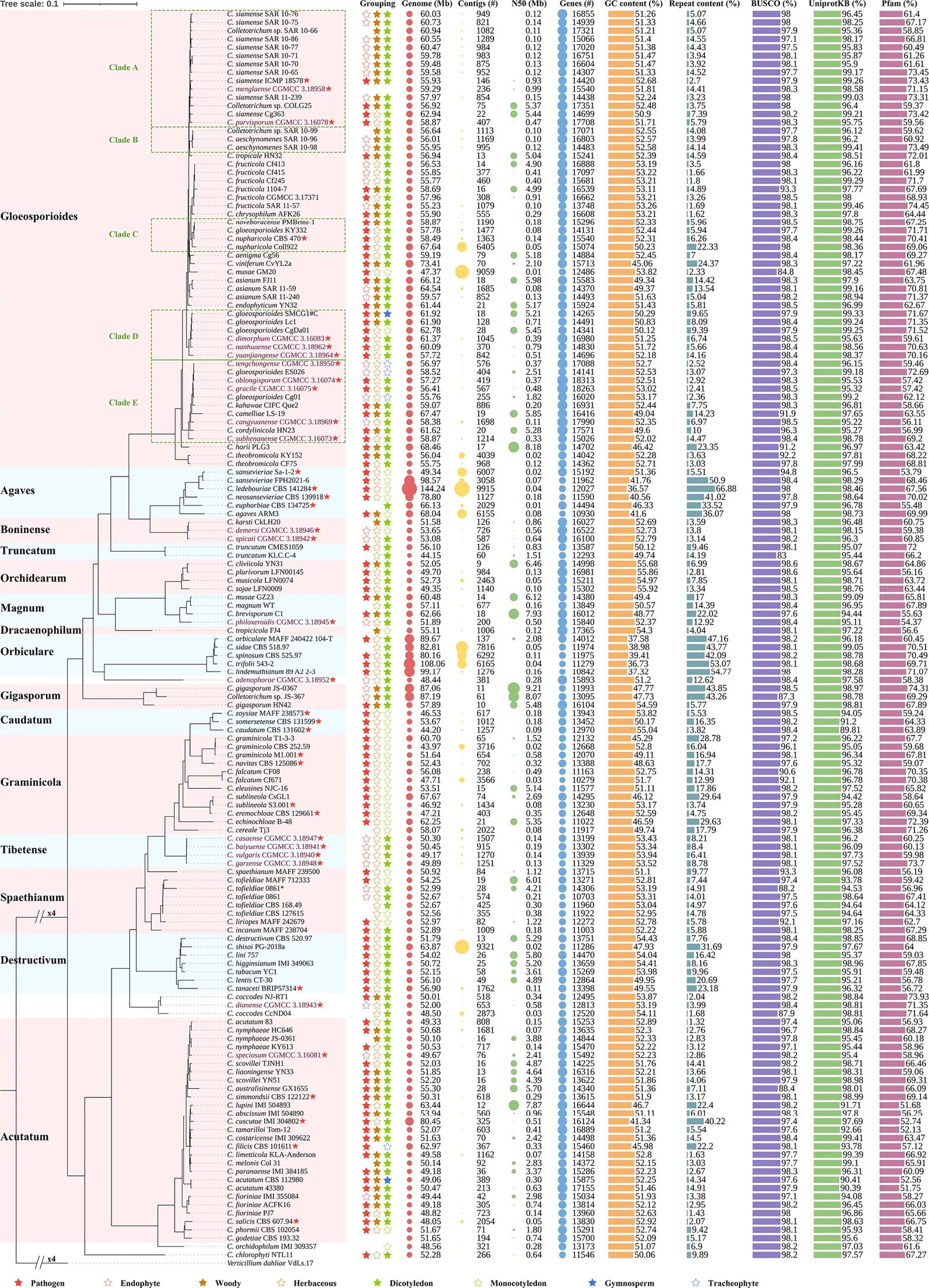
Phylogeny and metadata of the 150 genome-sequenced and analyzed *Colletotrichum* strains. Strains were ordered according to the phylogeny of 160 single-copy orthologous genes. Strain names highlighted in purple indicate those sequenced in this study. A red superscript pentagram indicates type strains of respective species. Grouping: Strains were grouped by lifestyle, host life-form and clade. Genome (Mb): Genome size. Contigs (#): The number of contigs/scaffolds. N50 (Mb): The length of the shortest contig needed to cover half of the total genome when contigs are ordered from longest to shortest. Genes (#): The number of predicted genes. GC content (%): The percentage of cytosine and guanine bases in genomes. Repeat content (%): The proportion of repeats in the genomes. BUSCO (%): Genome completeness by BUSCO assessment. UniportKB (%): The proportion of potentially functional protein-coding genes. Pfam (%): The proportion of protein-coding genes with at least one PFAM domain.

A total of 150 *Colletotrichum* genomes, representing 97 species from approximately 79 hosts, were analyzed, encompassing 15 species complexes and 4 singletons (Fig. 1). Phylogenomic reconstruction based on 160 single-copy orthologous genes, together with multilocus phylogenetic analysis (Suppl. material 1: fig. S1), consistently supported the stable taxonomic status of most species/strains, but also revealed certain misplacements. In Glosc, *C. menglaense* and *C. parvisporum*, along with two *Colletotrichum* sp. strains (SAR 10-66 and COLG25), clustered within a clade formed by *C. siamense* strains (Clade A). The multilocus phylogeny showed congruent results, while resolving clade A into multiple parallel subclades. *Colletotrichum* sp. SAR 10-99 was placed within the *C. aeschynomenes* clade (Clade B). Six strains labelled as *C. gloeosporioides* were distributed across three distinct clades (C, D, and E). Among them, the *C. gloeosporioides* reference strain SMCG1#C and type strain IMI 356878 (in the multilocus phylogeny) (Weir et al. 2012; Huang et al. 2019) resolved within clade D, so Lc1 and CgDa01 were correctly identified as this species. Meanwhile, *C. dimorphum* and *C. nanhuaense* also resolved within this clade, suggesting that both species may represent *C. gloeosporioides*. Strain KY332 in clade C and strains ES026 and Cg01 in clade E were misidentified as *C. gloeosporioides*.

In the singleton species, phylogenomic analysis revealed a close relationship between *C. dianense* and *C. coccodes* NJ-RT1, and multilocus phylogeny further placed them within the *C. nigrum* clade. Two database-derived strains, *Colletotrichum* sp. JS-367 and *C. acutatum* 83, were reassigned to *C. gigasporum* (in Gigsc) and *C. nymphaeae* (in Acusc), respectively. Additionally, topological incongruence was detected between phylogenomic and multilocus phylogenetic analyses for several species/strains. Multilocus phylogeny supported the conspecific status of *C. musae* GZ23 (in Glosc) and *C. liaoningense* YN33 (in *C. magnum* species complex; Magsc) with their respective type strains, whereas phylogenomic analysis placed their assemblies with *C. magnum* (in Magsc) and *C. scovillei* (in Acusc), respectively. Given that the genome-derived ITS sequences shared only approximately 87% identity with the previously reported sequences for these strains, this discordance likely reflects strain misidentification, sample mix-up, or erroneous accession annotation. Nevertheless, the combined evidence from the phylogenomic analysis, sequence similarity of the barcode loci, and their placement in the multilocus phylogeny indicated that the assemblies labelled as *C. musae* GZ23 and *C. liaoningense* YN33 most likely represent strains of *C. liaoningense* and *C. scovillei*, respectively (Suppl. material 3: table S5). Phylogenomic analysis also reclassified *C. vulgaris* from the Glosc to the Tibsc and placed singleton species *C. demersi* within the *C. boninense* species complex (Bonsc). These results further clarified phylogenetic relationships and corrected several previously misidentification strains in *Colletotrichum*, emphasizing the importance of phylogenomic analysis in resolving taxonomic classifications.

### Basic information and groups

Following the phylogenetic revision, genomic information of *Colletotrichum* species/strains was systematically analyzed. Genome size varied over a 3.3-fold range, from 43.97 Mb (*C. graminicola* CBS 252.59) to 144.24 Mb (*C. ledebouriae* CBS 141284), with most strains falling between 50 and 60 Mb (Fig. 1). GC content remained relatively stable (~52%) except for a few outliers. Repeat sequences (ReSeq) accounted for < 10% of the genome in 109/150 strains but exceeded 30% in 14 strains, reaching a maximum of 66.88% in *C. ledebouriae* CBS 141284. Strains with high ReSeq content generally exhibited lower assembly contiguity, through genome completeness remained unaffected. Despite the wide genome size variation, gene counts ranged from 10279 (*C. falcatum* Cf671) to 18313 (*C. oblongisporum* CGMCC 3.16074), showing no correlation with genome size (*R*^2^ = 0.02; *p* < 0.05). Of these genes, 89.81% to 99.46% were assigned UniProtKB annotations and 51.68% to 74.45% were classified by Pfam, indicating that most encoded proteins have known or predicted functions. These finding highlight substantial genomic diversity in *Colletotrichum*, particularly in genome size and ReSeq, while gene number remains relatively stable.

To elucidate genomic heterogeneity in *Colletotrichum*, strains were classified using a coarse scheme informed by isolation metadata, incorporating four dimensions: phylogeny, lifestyle, host clade and life-form (Suppl. material 1: fig. S2). Phylogenetic classification divided the strains into 16 groups, comprising 15 species complexes and a singleton group containing unassigned strains. Consistent with previous consensus, the widely distributed and abundant complexes Glosc and Acusc comprised 36.67% and 18.00% of the total, respectively, representing over half of the dataset (Talhinhas and Baroncelli 2021). However, Bonsc—the third most abundant species complex—has only limited genomic data available (sample size = 3). The rarely occurring *C. truncatum* (Trusc) and *C. dracaenophilum* (Drasc) species complexes were excluded from the comparative analyses due to insufficient sample sizes.

Grouping by host clade showed that *Colletotrichum* primarily infects dicotyledons (74.67%) and monocotyledons (20.00%), with fewer cases in tracheophytes and gymnosperms (4.00%) and unclassified hosts (1.33%) (Talhinhas and Baroncelli 2021). Based on lifestyle, the strains were predominantly classified as pathogens (65.34%), with a substantial proportion of endophytes (23.33%) (da Silva et al. 2020), and unknown status (11.33%). Additionally, the host plants infected by these strains were categorized by life-form as herbaceous (58.00%), woody (38.67%), or unknown (3.33%). Overall, the selected strains reflect the broad distributions of the known *Colletotrichum* species. Within categories, strains with the corresponding missing information were excluded from the comparative analysis.

### Genomic characteristics of *Colletotrichum*

**Figure 2.**
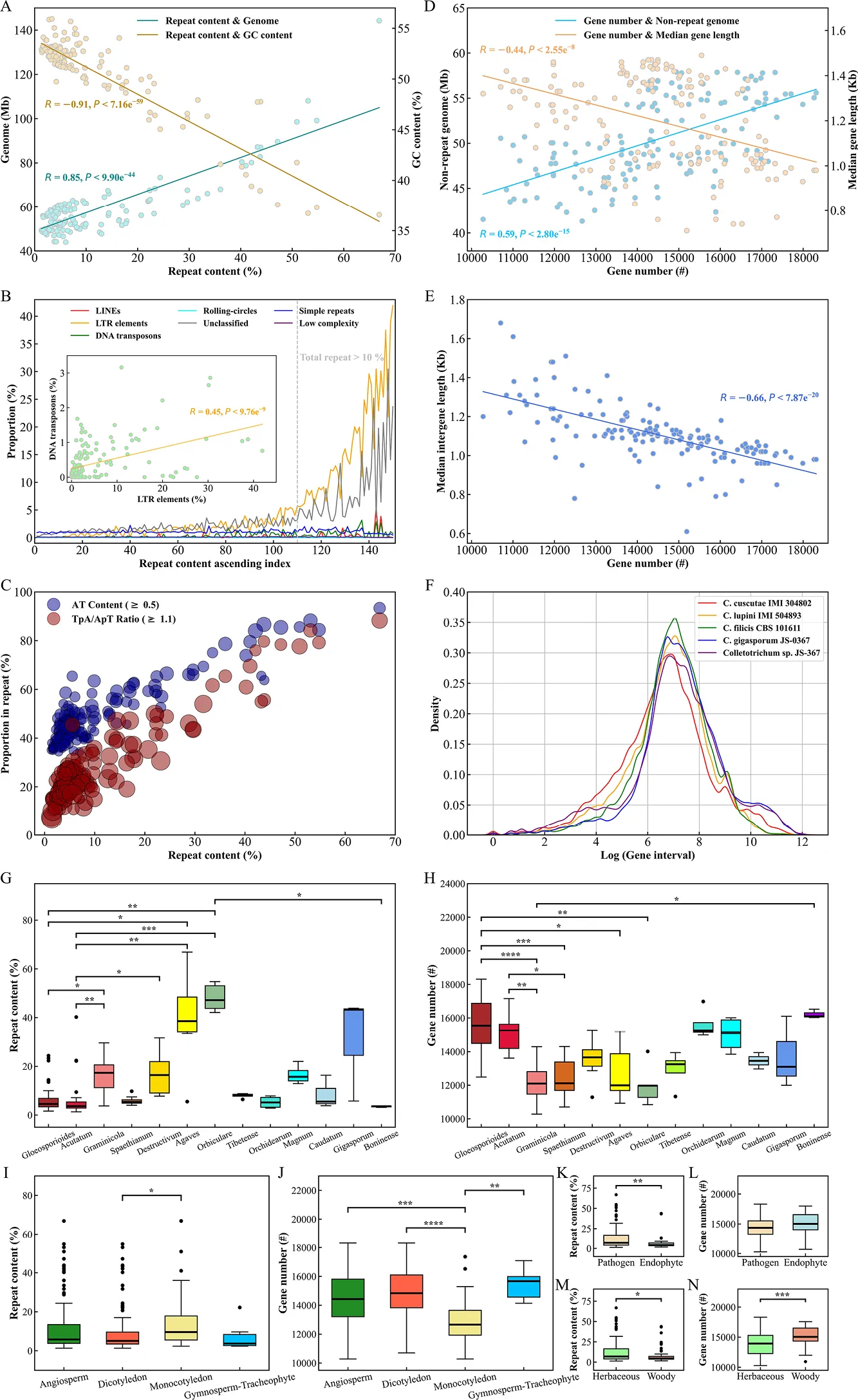
Comprehensive associations of genomic characteristics and intergroup comparisons of *Colletotrichum*. **A** Correlations of genome size and GC content with repeat content, respectively. **B** Proportion of various repetitive elements in the genome, with an inset showing the correlation between long terminal repeat (LTR) and DNA transposon content. **C** Proportion of repetitive sequences associated with high AT content and repeat-induced point mutation (RIP) occurrence. Bubble size represents the median value of AT/RIP in the corresponding repeat sequence set. **D** Correlations of non-repeat genome size and median gene length with gene number, respectively. **E** Correlations of median intergenic length with gene number. **F** Strains exhibiting a bimodal distribution of intergenic distance density, suggesting that they may possess a two-speed genome. **G–H** Difference in repeat content (**G**) and gene number **(H)** among 13 *Colletotrichum* species complexes. **I–J** Difference in repeat content (**I**) and gene number **(J)** among four host clade groups. **K–L** Differences in repeat content (**K**) and gene number **(L)** between pathogenic and endophytic *Colletotrichum*. **M–N** Differences in repeat content (**M**) and gene number **(N)** between hosts of herbaceous and woody plants. Levels of statistical significance were marked *, * *p* < 0.05, ** *p* < 0.01, *** *p* < 0.001, and **** *p* < 0.0001 (**G–M**).

Genomic analysis of *Colletotrichum* revealed a significant positive correlation between ReSeq content and genome size, and a significant negative correlation with GC content (*p* < 0.05; Fig. 2A), suggesting that ReSeq expansion may contribute to genome size variation and GC content reduction. Further analysis showed that genome enlargement is primarily associated with the amplification of LTRs and unclassified TEs, rather than DNA transposons. However, LTRs and DNA transposons exhibit a co-varying pattern (Fig. 2B), suggesting potential interactions between these elements.

RIP analysis indicated that genomes with higher ReSeq content also exhibit a higher frequency of RIP-associated mutations, accompanied by an increase in AT content and a corresponding decrease in GC content (Fig. 2C). Moreover, a direct comparison between genomes with low (< 30%) and high (> 30%) ReSeq content revealed a significant difference in mean RIP effect strength (1.63 vs 1.53; *p* < 0.05). The TE composition shifts and group-wise RIP differences suggested that inefficiency in the RIP defense mechanism against TEs may have facilitated significant genome expansion in *Colletotrichum*.

Gene number was positively correlated with non-repetitive genome size but negatively correlated with gene length (Fig. 2D), suggesting that gene number and length collectively influence genome architecture. Gene number was also strongly negatively correlated with intergenic spacing (Fig. 2E), implying that higher gene density is associated with reduced intergenic regions. Meanwhile, ReSeq content was negatively correlated with gene number (Suppl. material 1: fig. S3). The intergenic spacing distributions further suggested that strains in species such as *C. cuscutae*, *C. filicis*, *C. lupini*, and *C. gigasporum* may exhibit a distinct two-speed genome architectures (Fig. 2F). Overall, these findings indicate that ReSeq content influences *Colletotrichum* genome evolution by driving genome expansion, altering GC content via RIP activity, and impact gene number, length, and density.

Comparative analysis revealed significant differences in ReSeq content across *Colletotrichum* groups: phylogenetically, the broad-host Glosc and Acusc possessed significantly lower ReSeq content, whereas the narrow-host specialists (Grasc, *C. agaves* species complex (Agasc), Orbsc, and Dessc) exhibited substantially higher levels (*p* < 0.05; Fig. 2G); While strains associated with monocotyledons contain significantly more ReSeq than those colonizing dicotyledons (Fig. 2I). Pathogenic strains exhibit a marked increase in ReSeq content compared to endophytic strains (Fig. 2K), and strains from herbaceous plants possess significantly higher ReSeq content than those from woody hosts (Fig. 2M). Although ReSeq content varies significantly across groups, LTRs showed greater variation by phylogeny and host clades, whereas DNA transposons differed primarily by lifestyle and host life-form (Suppl. material 3: table S6), suggesting that distinct TEs types may play different roles in genomic evolution and adaptation. Additionally, gene number varied notably across *Colletotrichum* groups, with trends opposing those of ReSeq content (Fig. 2H, J, L, N), implying that the impact of ReSeq on the genome is evident even among groups.

### Codon usage bias

**Figure 3.**
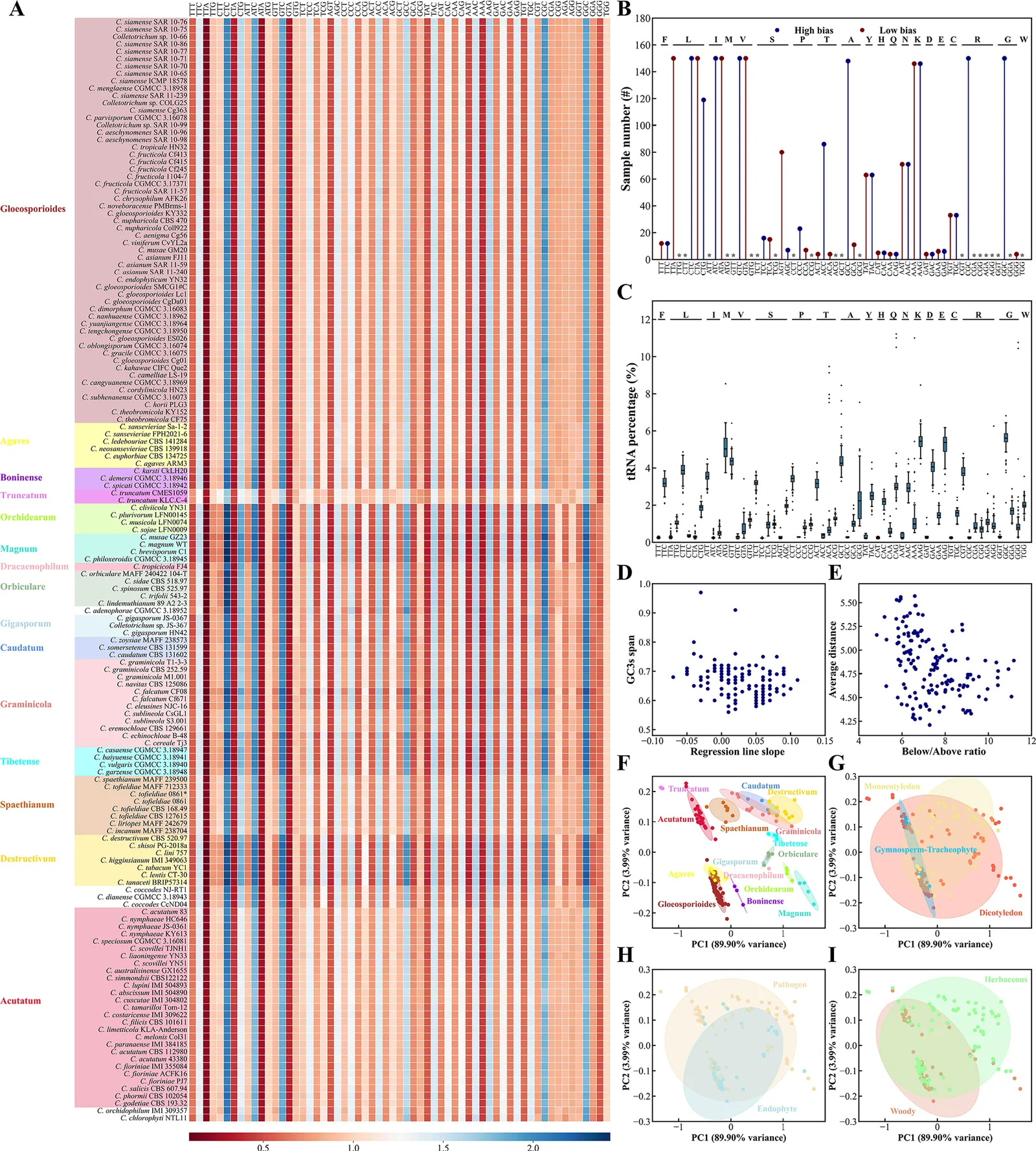
Codon usage bias and tRNA gene abundance in *Colletotrichum*. **A** Heatmap of relative synonymous codon usage (RSCU) distribution for 61 codons across all strains. **B** Number of strains exhibiting usage bias for each codon. RSCU value reflects the degree of usage bias. High bias (> 1.5), low bias (< 0.5). **C** Proportion of tRNA genes corresponding to each codon across all strains. **D** Distribution of regression line slope and GC3s spans derived from neutrality plot. **E** Distribution of the ratio of gene counts below/above the standard curve and the average distance of gene from the standard curve derived from the ENc plot. **F** Principal component analysis (PCA) of codon RSCU in the 13 *Colletotrichum* species complexes. **G**PCA of codon RSCU in three host clade group. **H**PCA of codon RSCU in pathogenic and endophytic *Colletotrichum*. **I**PCA of codon RSCU in hosts of herbaceous and woody plants. Each point in the scatter plot represents a *Colletotrichum* strain (**D–I**).

Codon usage bias (CUB) reflects selective constraints on translation efficiency and gene expression regulation, influencing genomic evolution and adaptation. As shown in Fig. 3, codon analysis of *Colletotrichum* revealed a pronounced CUB, characterized by a strong preference for cytosine at third codon positions (C3s) and a reduced frequency of adenine at third codon positions (A3s). Amino acid composition analysis indicated that codons encoding hydrophobic (L, I, V, and A) and positively charged (K and R) were more prone to exhibit bias (Fig. 3B). Neutrality plot analysis of 150 *Colletotrichum* strains showed a regression slope near zero (–0.07 to 0.11), alongside a broad distribution of GC3s values (Fig. 3D and Suppl. material 1: fig. S4), indicating that natural selection exerted a stronger influence than mutation in shaping CUB. The ENc plot further demonstrated a widespread deviation from the standard curve, with gene distribution primarily below it and a large average distance from the expected values (Fig. 3E and Suppl. material 1: fig. S5), reinforcing the dominance of selection over mutation. Despite the predominant role of natural selection, substantial heterogeneity in CUB pattern was observed across genes, suggesting that different genes or genomic regions were subject to varying degrees of mutational and selective pressures.

To investigate the relationship between CUB and tRNA availability in *Colletotrichum*, tRNA gene counts were compiled for each codon. The abundance of tRNAs corresponding to C3s and T3s exhibited a bimodal distribution, clustering at either high or low levels, whereas tRNAs for G3s remained at moderate levels, and those for A3s were consistently low (Fig. 3C). For twofold-degenerate codon families, tRNA gene copy number was generally associated with the magnitude of CUB: codon with low, moderate, and high tRNA gene copy numbers exhibited low, negligible, and high CUB, respectively (Fig. 3B, C and Suppl. material 1: fig. S6). The start codon ATG was an exception, showing a high tRNA gene copy number but no CUB. In codon families with multiple synonymous members, G3s and A3s codons maintain moderate tRNA gene counts regardless of CUB variations. Notably, except for glycine, all C3s codons exhibit extremely low or absent tRNA gene counts while displaying strong CUB. In contrast, T3s codons generally have abundant tRNA genes but show weak or negligible CUB (e.g., ACT). These discrepancies align with the Wobble hypothesis, which posits that the first anticodon position in tRNA enables non-strict base pairing at the third codon position, allowing flexible recognition. Overall, *Colletotrichum* exhibits distinct CUB-tRNA co-adaptation strategies: codons with two synonymous counterparts closely align with tRNA availability, whereas those with multiple synonyms exhibit stable or inverse correlations with tRNA gene counts, balancing translation efficiency and accuracy.

To assess RSCU variation among strains, principal component analysis (PCA) was conducted on 61 codons. PC1 and PC2 explained 89.90% and 3.99% of the total dispersion, respectively, with PC1 capturing the major axis of codon bias. Notably, 16 of the top 20 PC1 contributors were high bias codons (Suppl. material 1: fig. S7), indicating that variation in these codons largely shapes the codon usage pattern. Group-wise mapping revealed that codon usage profiles were highly concordant with phylogenetic distances, forming distinct clusters across species complexes (Fig. 1 and Fig. 3F). In contrast, other grouping schemes exhibited substantial overlap and, despite slight directional shifts, showed no clear separation between clusters (Fig. 3G–I). Consistently, the Mantel test further indicated that non-random usage bias that closely followed the phylogeny of *Colletotrichum* (*r* = 0.452, *p* = 0.001). However, Blomberg’s K test revealed marked heterogeneity in phylogenetic constraint among codons, with K values ranging from 0.079 to 3.291 (excluding ATG and TGG). Of the 59 codons, 16 (K > 1), 19 (0.5 ≤ K ≤ 1), and 24 (K < 0.5) were under strong, moderate, and weak phylogenetic constraint, respectively, with the latter more strongly influenced by external selective pressures (Suppl. material 3: table S7). Focusing on high bias codons under external selective pressures, comparison revealed that seven codons (AAG, CGC, GAG, TAC, TCC, TTC, and ATC) exhibited significantly higher usage bias in endophytic than in pathogenic strains (Suppl. material 3: table S8). TCC, AGC, and ATC were favored to a greater extent in strains infecting herbaceous (vs woody) hosts. In addition, strains associated with monocotyledons showed elevated preferences for AGC, CAC, and ATC compared with Gymnosperm–Tracheophyte-associated strains, whereas no significant differences were observed relative to strains associated with dicotyledonous. These findings suggested that codon usage patterns in *Colletotrichum* are primarily shaped by phylogenetic history, while lifestyle- and host-associated differences contribute to selective bias in a subset of codons.

### Composition of functional gene repertoires

**Figure 4.**
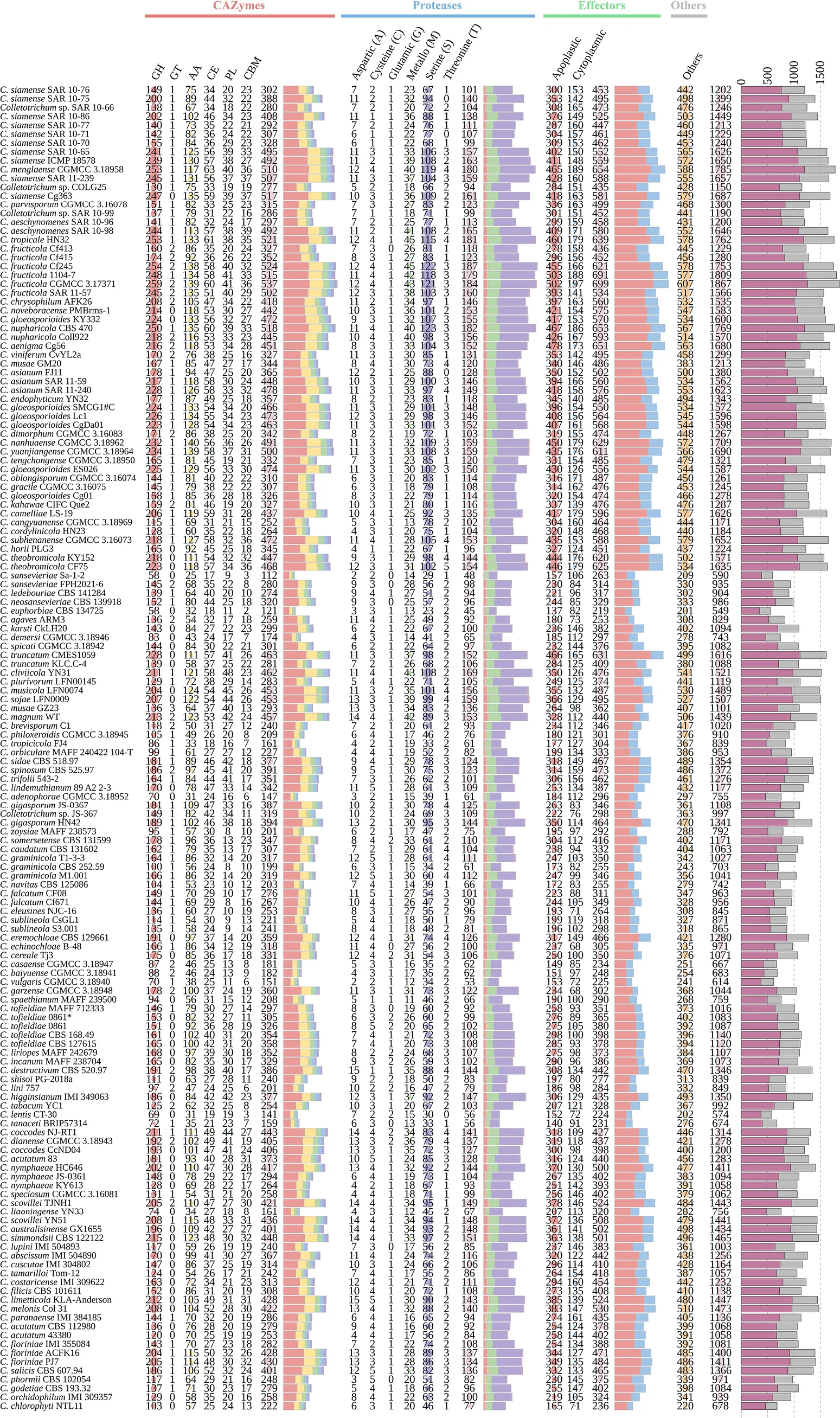
The secretome composition of 150 *Colletotrichum* strains. The categories presented from left to right are CAZymes, proteases, effectors, and others. Circle size represents quantity, with the total displayed as a cumulative bar chart. GH: glycoside hydrolases. GT: glycosyltransferases. AA: auxiliary activities. CE: carbohydrate esterases. PL: polysaccharide lyases. CBM: carbohydrate-binding modules.

Secreted proteins are essential for fungal–host interactions. The *Colletotrichum* secretome, defined as proteins containing signal peptides but lacking transmembrane helices, was mainly classified into CAZymes, proteases, effectors, and others. As shown in Fig. 4, secretome size varied markedly even among closely related strains, ranging from 549 (*C. euphorbiae* CBS 134725) to 1,867 (*C. fructicola* CGMCC 3.17371), with the secretome proportion ranging from 3.79% (*C. euphorbiae* CBS 134725) to 11.56% (*C. tropicale* HN32) of the total proteome and showing a weak positive correlation with proteome size (*R* = 0.23, *p* < 0.01). Effectors (219–699) and other secreted proteins (201–607) constituted the major components of the secretome, followed by CAZymes (112–537), whereas proteases (45–187) were consistently less abundant. Overall, the abundance of individual secreted components was strongly positively correlated with total secretome size across strains (Suppl. material 1: fig. S8). Nevertheless, coefficients of variation indicated lower inter-strain variability in effectors and other secreted proteins (0.259 and 0.219) than in CAZymes and proteases (0.297 and 0.277).

Comparative analyses further revealed pronounced lineage- and host-associated differentiation in secretome size (Suppl. material 1: fig. S9). Among the 13 species complexes, Glosc and Orcsc possessed substantially larger secretomes, whereas Agasc, Causc, Grasc, Tibsc, and Dessc exhibited markedly reduced secretome sizes, with significant differences observed between these contrasting groups. Notably, although Acusc had significantly smaller secretomes than Glosc, it still maintained significantly higher abundance than Agasc and Grasc. In addition, strains associated with woody or dicotyledonous hosts possessed larger secretomes than those infecting herbaceous or monocotyledonous hosts, whereas no significant differences were detected between pathogenic and endophytic strains. Taken together, broad host-range lineages such as Glosc and Acusc—predominantly associated with dicotyledonous woody plants—possessed larger secretomes, whereas lineages with high specificity toward monocotyledonous (Agasc, Causc, Grasc) or dicotyledonous (Tibsc and Dessc) herbaceous hosts exhibited reduced secretome repertoires. These findings suggested that secretome expansion is associated with infection of dicotyledonous woody hosts and with lineage-level host-range broadening.

**Figure 5.**
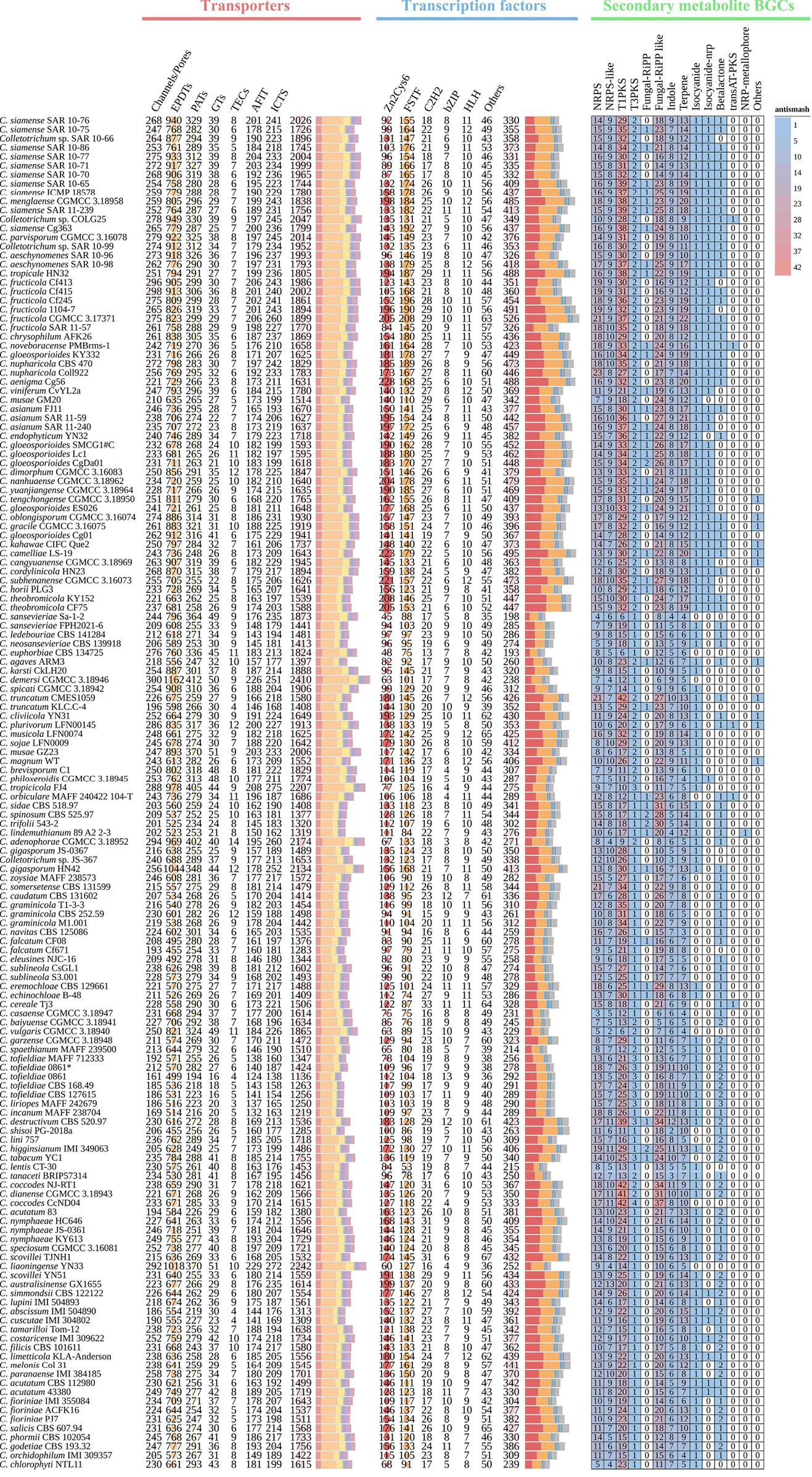
Information on transporters, transcription factors, and secondary metabolite biosynthetic gene clusters in the genomes of 150 *Colletotrichum* strains. Circle size represents quantity, with the total displayed as a cumulative bar chart. EPDTs: electrochemical potential-driven transporters. PATs: primary active transporters. GTs: group translocators. TECs: transmembrane electron carriers. AFIT: accessory factors involved in transport. ICTS: incompletely characterized transport systems. FSTF: fungal specific transcription factor. NRPS: non-ribosomal peptide synthetase. T1PKS: type I polyketide synthase. T3PKS: type III polyketide synthase. Fugal-RiPP: fungal ribosomally synthesized and post-translationally modified peptides. Isocyanide-nrp: isocyanide non-ribosomal peptide. transAT-PKS: trans-acting polyketide synthases. NRP-metallophore: non-ribosomal peptide metallophore.

Beyond the secretome, transporters, transcription factors, and SMBGCs also play important roles in fungal survival and in sensing and adapting to host-associated environments. In *Colletotrichum*, transporter repertoires ranged from 1,136 (*C. tofieldiae* 0861) to 2,410 (*C. demersi* CGMCC 3.18946) (Fig. 5). Electrochemical potential-driven transporters (EPDTs), mainly involved in nutrient and small-molecule uptake, represented the dominant class, followed by channels/pores and primary active transporters (PATs), whereas other classes occurred at lower levels. In contrast, transcription factors were present in lower numbers in *Colletotrichum*, ranging from 193 (*C. euphorbiae* CBS 134725) to 526 (*C. fructicola* CGMCC 3.17371). Across all 150 strains, a total of 43 transcription factor families were identified, with Zn_2_Cys_6_-type and fungi-specific transcription factors (FSTFs) representing the predominant families, followed by C2H2-, bZIP-, and helix-loop-helix (HLH)-type transcription factors. The remaining families were diverse, but rare.

*Colletotrichum* possessed considerable biosynthetic potential for secondary metabolites, with SMBGCs ranging from 32 (*C. vulgaris* CGMCC 3.18940) to 133 (*C. destructivum* CBS 520.97). These clusters encoded the major classes of secondary metabolites, including non-ribosomal peptides (NRPs), RiPPs), type I polyketide synthases (T1PKSs), terpenes, and indoles. These metabolite classes encompass compounds with diverse ecological and biological functions, including the modulation of plant immunity and disease development, as well as antimicrobial, antifungal, and hormone-related activities (Moraga et al. 2018; Kim and Shim 2019).

Consistent with the secretome trends, group-wise comparison revealed that Glosc exhibited markedly higher abundances of these functional repertoires than most other species complexes, particularly for SMBGCs (Suppl. material 1: fig. S9). Among the remaining lineages, transporter numbers were larger in Bonsc and Magsc, and transcription factor repertoires were relatively abundant in Acusc and Orcsc. Conversely, more host-specialized lineages (Agasc, Orbsc, Causc, Grasc, Tibsc, Spasc, and Dessc) generally exhibited lower abundances across these functional repertoires, to varying degrees. Across ecological groups, endophytic strains showed significantly higher transporter abundances than pathogenic strains, while strains associated with woody or dicotyledonous hosts exhibited larger transport, transcription factors and SMBGC repertoires compared with herbaceous or monocotyledonous hosts. Together with the secretome, these functional repertoires may collectively contribute to adaptation to dicotyledonous woody hosts and lineage-level host-range expansion.

**Figure 6.**
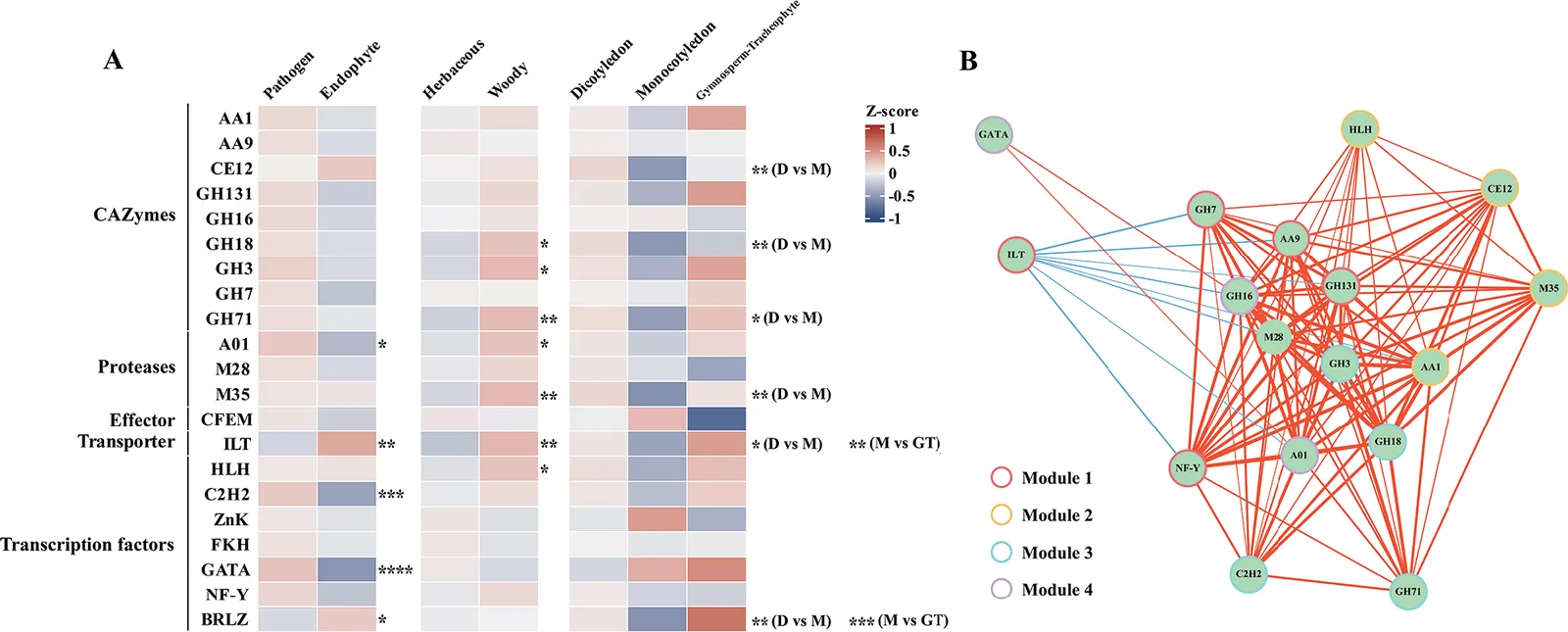
Ecological differentiation and co-occurrence patterns of functional categories with weak phylogenetic signal. **A** Heatmap showing Z-scored-standardized gene abundances of the 21 functional categories with weak or negligible phylogenetic signal (Pagel’s λ < 0.4) across ecological groups. **B** Co-occurrence network constructed from the same 21 categories based on pairwise abundance correlations across 150 *Colletotrichum* strains. Nodes represent functional categories, and edges denote significant co-occurrence relationships; red and blue edges indicate positive and negative correlations, respectively, with only associations having correlation coefficients ≥ 0.4 or ≤ −0.4 shown. CFEM: common in fungal extracellular membrane effectors (PF05730). ILT: iron/lead transporter (2.A.108). HLH: helix-loop-helix (PF00010). C_2_H_2_: Cys2-His2-type (PF00096). ZnK: zinc knuckle (PF00098). FKH: forkhead domain (PF00250). GATA: GATA-type (PF00320). NF-Y: histone-like transcription factor (PF00808). BRLZ: basic region leucine zipper (PF07716). Levels of statistical significance were marked with star *, * *p* < 0.05, ** *p* < 0.01, and *** *p* < 0.001 (**A**).

By integrating information on strain-level prevalence, interspecific variability, and relative abundance, we identified 81 major functional categories exhibiting high variability within the functional gene repertoire of *Colletotrichum* (Suppl. material 3: table S9). Pagel’s λ assessment indicated that most categories exhibited moderate to strong phylogenetic dependence, whereas only 21 displayed weak or negligible phylogenetic signal (λ < 0.4). Comparisons across ecological groups revealed that these categories were generally more abundant in pathogenic strains and in strains infecting woody or dicotyledonous hosts (Fig. 6A).

Gene co-occurrence network analysis further uncovered extensive coordinated associations among these categories, with clustering delineating four putative modules potentially linked to ecological adaptation: Module 1 (AA9, GH131, GH7, ILT [iron/lead transporter; 2.A.108], and NF-Y[histone-like transcription factor; PF00808]), Module 2 (AA1, CE12, M35, and HLH [PF00010]), Module 3 (GH18, GH3, GH71, and C2H2 [PF00096]), and Module 4 (GH16, A01, and GATA [PF000320]) (Fig. 6B). Among these, Module 1, mainly composed of cellulose degradation enzymes, showed minimal variation across ecological groups, except for ILT, which displayed significant intergroup differences. In contrast, Module 2, primarily involved in host cell wall structural breakdown (modification and degradation), and Module 3, associated with fungal cell wall remodeling, showed significantly higher abundance in strains infecting woody or dicotyledonous hosts. Most categories within Module 4 were significantly more abundant in pathogenic strains than in endophytic strains, particularly GATA, suggesting a tight association with pathogenicity. Despite not forming a distinct module, BRLZ (basic region leucine zipper; PF07716) showed significantly elevated abundance in endophytic strains and reduced in those infecting monocotyledon hosts, indicating a potential role in ecological adaptation.

### Gene family expansion/contraction across evolutionary history

**Figure 7.**
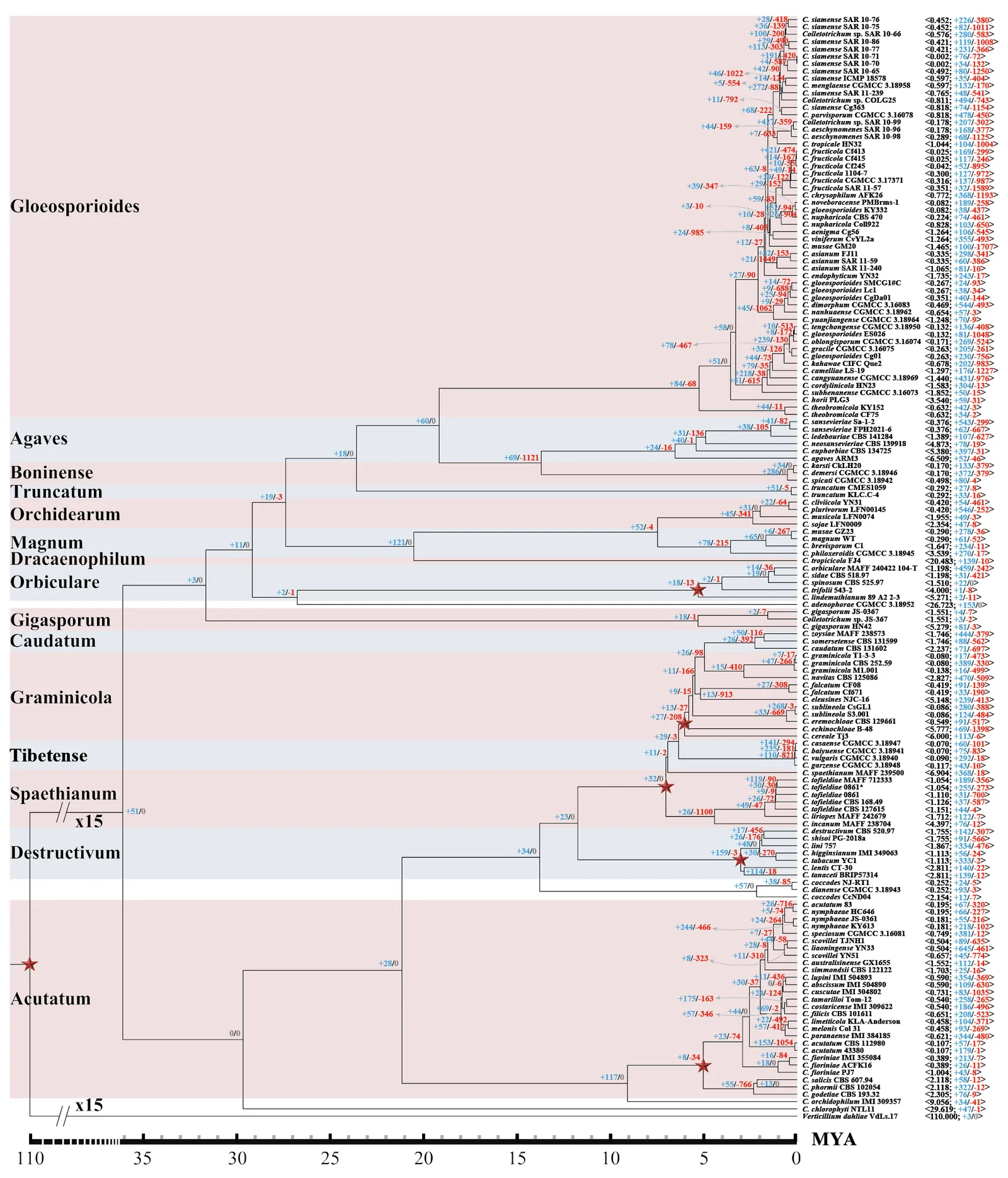
Gene family expansion and contraction of 150 *Colletotrichum* strains. The Maximum Likelihood tree was constructed based on a combined dataset by concatenating 160 single-copy proteins. Calibration nodes were noted using red stars. Node age corresponded to the timeline below, and divergence times are shown in millions of years. The number of significantly expanded (blue) and contracted (red) gene families (*p* < 0.05), along with those showing no change (gray) are given on the corresponding branches. Node age and gene family change for species/strains are indicated in angle brackets. MYA: million years ago.

The ML tree constructed from tandem gene families, incorporating six calibration nodes, was used to infer the divergence time of *Colletotrichum* (Fig. 7). The evolutionary timeline indicated that the most recent common ancestor (MRCA) of *Colletotrichum* emerged approximately 36.06 million years ago (MYA), diverging into two major branches. Most species complexes were estimated to have diverged between 5 and 10 MYA, with 59.33% of species/strains forming within the last 1 MYA. While the MRCA of the Glosc aligned with previous estimates, other species’ complexes appeared younger than previously reported (Liang et al. 2018; Bhunjun et al. 2021; Chen et al. 2022). Notably, the MRCAs of Bonsc, Trusc, and Tibsc were exceptionally recent, emerging within the past 1 MYA. These discrepancies arose from updated calibration points, which have shifted divergence estimates to more recent periods. For example, the divergence estimates for Dessc, Spasc, and Grasc have been revised from 10–20 MYA in earlier studies (Chen et al. 2022) to 3–7 MYA based on corrections to the underlying Timetree databases. Additionally, strain selection differences—such as insufficient sampling or the inclusion of closely related strains—skewed the MRCA estimates of these species’ complexes toward younger ages. Nonetheless, the updated evolutionary timeline suggested that *Colletotrichum*’s formation and diversification occurred more recently than previously suggested, providing a refined framework for understanding its evolutionary history.

A total of 64,174 gene families were identified across 150 *Colletotrichum* strains and an outgroup (*Verticillium dahliae* VdLs.17), among which 3,771 exhibited significant changes (*p* < 0.05) across the phylogeny, including 1,852 contractions and 1,919 families showing both expansion and contraction (Suppl. material 3: table S10). GO slim classification revealed that most families were associated with enzyme-related catalytic activities (GO: 0003824), with hydrolase, transferase, and oxidoreductase activities representing the most abundant categories, together with a substantial contribution from transporter activity (GO:0005215) and transmembrane transport (GO:0055085). In contrast, functions related to transcription regulation (GO:0140110) and core metabolic processes (GO:0006629, GO:0005975, and GO:0006520) were less represented, and cellular structure and development were comparatively underrepresented, suggesting that variations in metabolic and transport capacities may have play a major role in speciation and strain diversification within *Colletotrichum* (Suppl. material 3: table S11). Compared with *V. dahliae*, 51 gene families were significantly expanded in *Colletotrichum*, predominantly involving oxidoreductases and hydrolases, secondary metabolism-associated enzymes, MFS-type transporters, and Zn_2_Cys_6_-type transcription factors, indicating enhanced metabolic versatility, environmental adaptability, and host interaction potential (Suppl. material 3: table S12). Notably, only limited expansions occurred before the emergence of complex species, while extensive gene family changes arose during their formation and subsequent diversification (Fig. 7).

**Figure 8.**
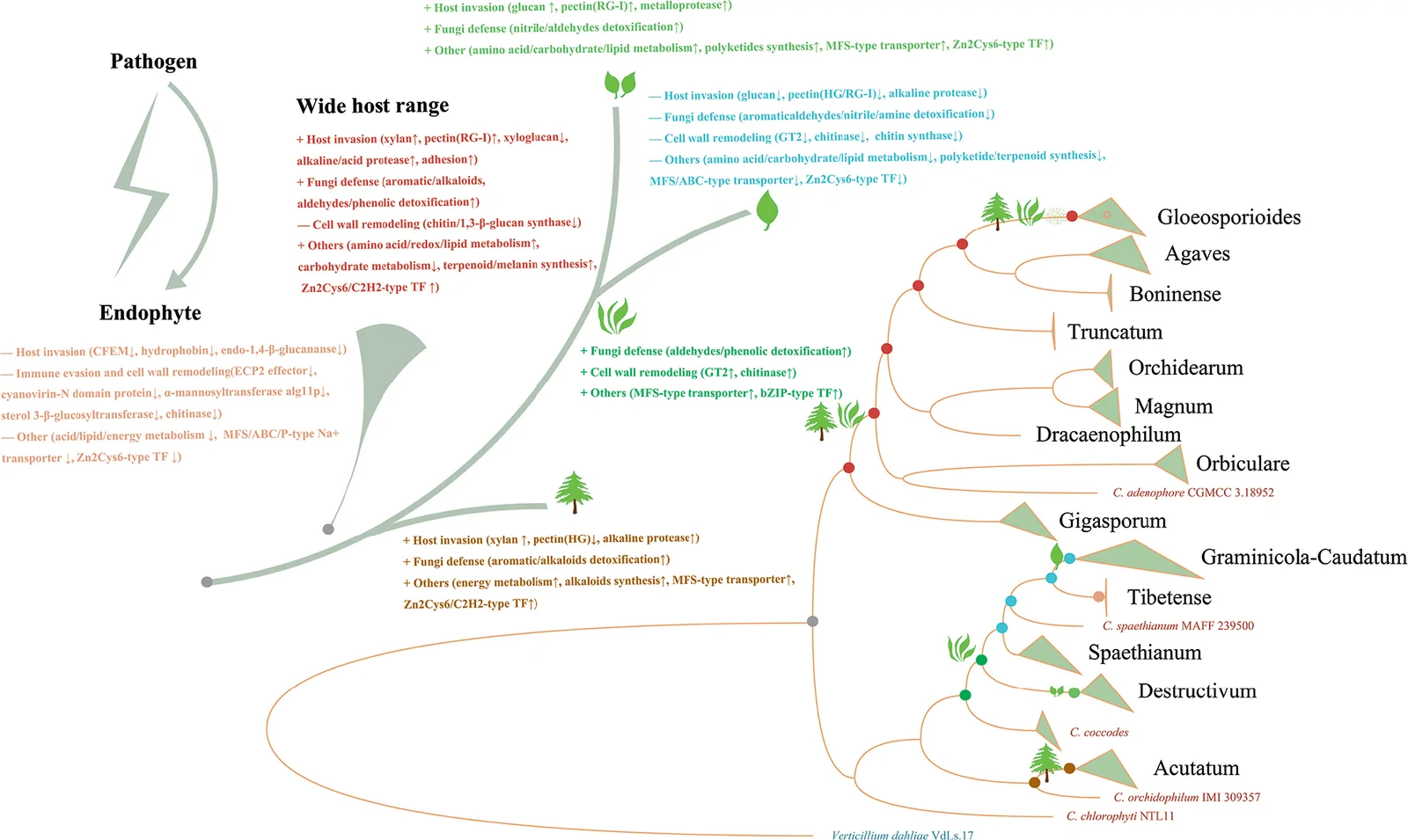
Schematic representation of lineage-specific adaptive strategies in *Colletotrichum*. Solid circles on the phylogeny indicate nodes used for lineage diversification analyses, whereas open circles represent nodes of internal diversification within species complexes. Two internal nodes were analyzed: siamense_endophyte (*C. siamense* SAR 10-76, *C. siamense* SAR 10-75, *Colletotrichum* sp. SAR 10-66, *C. siamense* SAR 10-86, *C. siamense* SAR 10-77, *C. siamense* SAR 10-71, *C. siamense* SAR 10-70, and *C. siamense* SAR 10-65), and fructicola_endophyte (*C. fructicola* Cf413, *C. fructicola* Cf415, and *C. fructicola* Cf245). Significantly expanded/contracted gene families associated with host invasion, fungi defense and detoxification, immune evasion and cell wall remodeling, core metabolism, secondary metabolism, transport processes, and transcriptional regulation were analyzed.

To elucidate the major features underlying shifts in ecological adaptation, several key evolutionary nodes were examined (Fig. 8). During the formation of the woody host-dominant lineage (Acusc), host infection-associated genes showed pronounced expansion of GH39 and alkaline proteases, accompanied by contraction of pectate lyase. Detoxification-related genes also expanded, particularly those targeting aromatic and alkaloid compounds, including cytochrome P450 monooxygenases, FAD-dependent monooxygenases, and 2,6-dihydropseudooxynicotine hydrolases. Moreover, genes involved in energy metabolism and alkaloid biosynthesis were also expanded (Fig. 8 and Suppl. material 3: table S13). In contrast, the emergence of the herbaceous host-specific lineage was characterized by expansion of detoxification genes targeting reactive small molecules (phenolic and aldehyde compounds), such as laccases, aldo/keto reductases, and glutathione-dependent formaldehyde-activating enzymes. GT2 and chitinase families involved in cell wall remodeling were likewise expanded.

Subsequent divergence within herbaceous host-associated lineages revealed distinct evolutionary trajectories between dicot- and monocot-infecting clades. The dicot-specific lineage (Dessc) showed expansion of gene involved in glucan (β-1,3-endo glucanase) and pectin (arabinan endo-1,5-α-L-arabinosidase) degradation, along with nitrile and aldehyde detoxification. By contrast, the monocot-specific lineage (Grasc and Causc) underwent pronounced contraction of these corresponding gene families, including glucanases, pectate lyases, rhamnogalacturonan endolyases, nitrilases and aldehyde dehydrogenases. This lineage also displayed extensive contraction of genes related to cell wall remodeling, detoxification of aromatic and amine compounds, core metabolism, secondary metabolite biosynthesis, MFS/ABC-type transporters, and Zn_2_Cys_6_-type transcription factors, indicating substantial narrowing of functional gene repertoires.

During the formation of the broad host-range lineage (Glosc), both the GH39 and the exo-rhamnogalacturonase were expanded, whereas α-L-arabinofuranosidase and xyloglucanase showed contraction. Coordinated expansion of acid and alkaline proteases with complementary pH preferences was also observed, together with adhesion-related proteins. Detoxification-related genes likewise expanded broadly, targeting aromatic and alkaloid compounds as well as reactive small molecules (phenolics and aldehydes), whereas genes associated with cell wall biosynthesis were reduced. Additional expansion included genes involved in core metabolism, terpenoid and melanin biosynthesis, and Zn_2_Cys_6_ and C_2_H_2_ transcription factors, while carbohydrate metabolism-related gene families represented a notable exception and showed contraction. For endophytic lineages, a common trend was the pronounced contraction of gene families associated with adhesion-related proteins (CFEM and hydrophobins) and endo-1,4-β-glucananse in host invasion, as well as those involved in cell wall remodeling (e.g., chitinase and sterol 3-β-glucosyltransferase). Beyond these changes, concurrent contractions in amino acid, lipid, and energy metabolism, along with various transporters and transcription factors families, indicated functional simplification associated with an endophytic lifestyle.

## Discussion

Currently, species identification in *Colletotrichum* has mainly relied on multilocus phylogenies and morphological characters, yet both approaches have limitations in distinguishing closely related taxa. Morphological traits are highly plastic and often overlapping among species, while molecular data may be compromised by database inaccuracies and variable marker resolution (Cannon et al. 2008; Cai et al. 2009; Crouch et al. 2009a; Cannon et al. 2012). Recent studies indicated that phylogenomics provide higher resolution and taxonomic accuracy than traditional multilocus sequence typing (MLST) (Menicucci et al. 2025). In this study, both phylogenomic and MLST results are consistent with the proposed synonymization of *C. menglaense* and *C. parvisporum* with *C. siamense*, and *C. dimorphum* and *C. nanhuaense* with *C. gloeosporioides*, as suggested by previous taxonomic revisions (Zhang et al. 2023a; Zhang et al. 2023b; Sui et al. 2024). Although phylogenomic evidence is lacking, available MLST analyses favor treating *C. dianense* as a synonym of *C. nigrum* (Yarmeeva et al. 2023; Chang et al. 2024). These misidentified species likely resulted from initial classifications based on single representative strains without sufficient clade-level support, leading to biased species inference. Therefore, the taxonomic status of these taxa requires further validation through expanded sampling and integrated genomic, multilocus, and morphological evidence (Xu et al. 2016; Xu 2020). The reassignment of several strains revealed pervasive taxonomic misannotations in public genomic datasets, which may result in misleading interpretations in comparative genomic analyses. This issue is particularly relevant to the endophytic *Colletotrichum* strains ES026 and Cg01, which are known for huperzine production and have been widely used as *C. gloeosporioides* in previous studies (Shu et al. 2014; Kang et al. 2019). However, phylogenomic and MLST analyses strongly suggested that they form distinct lineages and may represent two putative novel species, warranting further polyphasic taxonomic assessment.

Topological incongruence between phylogenomic and MLST analyses was observed in few species/strains. These conflicts indicate that similarity at conventional marker loci may not always reflect genome-wide evolutionary relationships, possibly due to limited phylogenetic signal or locus-specific evolutionary histories. Although multilocus phylogeny remains useful for comparison with existing taxonomic frameworks, genome-scale evidence provides a more comprehensive basis for reassessing unstable taxa. Our revised classifications thus provide a more reliable framework for accurate genomic interpretation of *Colletotrichum* in the post-genomic era.

Fungal genome sizes vary widely, spanning from under 3 Mb to over 100 Mb (Stukenbrock and Croll 2014). In this study, *Colletotrichum* genomes exhibit substantial variation (43.97~144.24 Mb), exceeding the previously reported ranges of Chen et al. (2022) (44.15~109.66 Mb) and Liu et al. (2022) (35.03~109.66 Mb). The exceptionally small genome of *C. godetiae* C184 (35.03 Mb), with a completeness score below 80%, likely represents an incomplete assembly. In Liu’s study (2022), the next smallest genome, *C. paraendophytum* (43.96 Mb), is part of the Grasc alongside *C. graminicola* CBS 252.59 (43.97 Mb). These findings imply that minimal genome in *Colletotrichum* is usually associated with specific infection of monocot plants.

Repeat sequences, particularly TEs, significantly shape fungal genome structure and size (Möller and Stukenbrock 2017; Mat Razali et al. 2019), typically accounting for 0% to 30% of genome, a pattern also observed in *Colletotrichum* (Castanera et al. 2016). Consistent with Chen et al. (2022), TE content was a primary determinant of genome size and GC content in *Colletotrichum* species/strains. We found that reduced RIP efficiency may permit greater TE accumulation, contributing to the development of larger *Colletotrichum* genomes. Comparative analysis of TEs revealed significant differences at the species level and above, with TE content and gene numbers complementing each other, reducing overlap within the genome (Muszewska et al. 2019). However, exceptions existed, such as the Grasc and Dessc, which exhibited both high repeat sequence content and gene numbers, suggesting TEs may exert a distinct influence on gene regulation. Additionally, TE patterns varied among strains with different lifestyle and host origins, being more prevalent in pathogenic strains and those from monocot herbaceous plants (Muszewska et al. 2019; Baroncelli et al. 2024). Thus, while lineage-specific evolutionary history may partly account for TE variation among species, ecological niche also plays an important role.

Codon usage bias (CUB) is widespread in biological systems (Masłowska-Górnicz et al. 2022; Parvathy et al. 2022; Wint et al. 2022). In *Colletotrichum*, despite TE amplification disrupting genomic GC content, a consistent preference for C3s and avoidance of T3s was observed across species. Fungal pathogens often align their codon usage with that of host plants (Gupta and Singh 2021; Nisa et al. 2024), but *Colletotrichum* deviates from this trend. Monocots typically favor G/C-ending codons, whereas dicots prefer A/U-ending codons (Wang and Roossinck 2006). In contrast, *Colletotrichum* consistently exhibits a preference for G/C-ending codons, despite its association with dicot hosts. One possible reason is that the broad host adaptability of these species weakens the link between their codon usage and the host, as generalist lifestyles are also associated with enhanced codon optimization (Badet et al. 2017).

PCA and Mantel test analyses indicated that CUB in *Colletotrichum* is largely structured by phylogenetic history, with patterns primarily reflecting evolutionary relationships rather than lifestyle or host type. Nevertheless, ecological factors can still exert selective pressures on the usage of specific codons. Neutrality and ENc analyses further support natural selection as the main force shaping CUB, with substantial heterogeneity among genes within species. Collectively, CUB in *Colletotrichum* is primarily driven by natural selection, constrained by phylogenetic conservation, and fine-tuned by ecological adaptation. Evidence from numerous studies indicates that CUB and the abundance of corresponding tRNAs are closely correlated, reflecting co-evolution between of the two (Rocha 2004; Behura and Severson 2011; Badet et al. 2017).

In *Colletotrichum*, we observe a bistable co-evolutionary relationship between CUB and tRNAs, consistent with Higgs’s prediction that codon usage and tRNA gene content co-evolve into mutually adaptive states, often stabilizing in multiple equilibria (Higgs and Ran 2008). The inconsistencies observed in multi-synonymous codon can be explained by the “wobble hypothesis”, as adenine (A) at the first position of tRNA anticodons (the wobble site) is rarely observed due to its modification to inosine (I) (Schmitt et al. 2024), which facilitates stable wobble pairing with three different bases—a phenomenon common in bacteria and fungi (Rafels-Ybern et al. 2018; Rafels-Ybern et al. 2019). Moreover, the eight inconsistencies observed in *Colletotrichum* align with the typical number of inosine-modified tRNAs in eukaryotes (Rafels-Ybern et al. 2018; Rafels-Ybern et al. 2019), suggesting that *Colletotrichum* adopts a dual strategy: strict codon-anticodon pairing and wobble pairing via A-to-I modification. This strategy likely reflects an evolutionary balance optimizing both translational efficiency and accuracy.

The fungal secretome constitutes a key molecular interface between fungi and plant hosts, underpinning processes involved in infection, colonization, immune suppression, and virulence. Previous work reported contractions of secreted CAZyme and protease families in narrow-host-range and monocot-associated *Colletotrichum* lineages (Baroncelli et al. 2016; Baroncelli et al. 2024). Our results showed that lineages with broadly woody dicot hosts (Glosc and Acusc) have significantly larger secretomes than host-specialized lineages infecting herbaceous monocot (Grasc, Causc and Agasc) or dicot hosts (Dessc and Tibsc). Abundant secreted CAZymes may enhance their ability to cope with complex woody and dicot host cell walls, which are characterized by higher hemicellulose and lignin contents in woody plants and greater pectin abundance in herbaceous dicots than in monocots (Vogel 2008; Vassilev et al. 2012; Haas and Peaucelle 2021; Birkeland et al. 2025). Meanwhile, the enrichment of secreted serine, metallo-, and cysteine proteases, adapted to self-induced alkalinization of host tissues, facilitates effective penetration by degrading structural and defense-related proteins (Shnaiderman et al. 2013; Fernandes et al. 2017); whereas monocot type II cell walls are typically protein-poor (Calderan-Rodrigues et al. 2019; Baez et al. 2022), and strains infecting these hosts consequently possess fewer secreted proteases.

Although variation in immune responsiveness among plant types remains poorly understood, *Colletotrichum* strains infecting woody dicot hosts show increased effector repertoires, suggesting stronger selection for immune suppression. Additionally, the coordinated expansion of the secretome and other functional repertoires (transporters, transcription factors, and SMBGCs), reflects a system-wide genomic adaptation associated with host type. However, Krijger et al. (2014) pointed out that the composition of the fungal secretome is largely influence by phylogenetic history. Here, we also observed strong phylogenetic signals across most functional gene categories in *Colletotrichum*, while several co-occurring functional modules potentially represent signatures of ecological selection. Modules related to fungal cell wall remodeling (Module 3) and host cell wall disruption (Module 2) were enriched in strains infecting woody and dicot hosts. These modules include chitinases (GH18) and β-glucanases (GH71) involved in immune suppression (Yang et al. 2019; Liu et al. 2023; Gu et al. 2024), as well as enzymes mediating lignin oxidation, pectin modification, and protein degradation (AA1, CE12, and M35), suggesting selective pressures imposed by host cell wall complexity and immune responses. In addition, GH16, A01, and GATA in Module 4 were significantly enriched in pathogenic strains relative to endophytes, highlighting their potential roles in pathogenic lifestyles. Notably, GATA transcription factors such as NsdD and AreA have previously been implicated in the virulence of *Colletotrichum* (Bi et al. 2017; Wang et al. 2025). Overall, functional gene diversity in *Colletotrichum* emerges from a hierarchy of strong phylogenetic constraints overlaid by host- and lifestyle-driven ecological selection.

Gene loss is a major force in genome evolution (Albalat and Cañestro 2016). Across *Colletotrichum* evolutionary trajectories, significantly contracted gene families greatly exceeded expanded ones. Similarly, gene families located near simple repeat sequences are likely influenced by RIP effects (Suppl. material 1: fig. S10), which limit local duplication and expansion (Hane et al. 2015). However, host range and type have been linked to the expansion of specific functional gene families (Baroncelli et al. 2016; Baroncelli et al. 2024). Thus, *Colletotrichum* genomes appear to be shaped by a dynamic balance among neutral gene loss, RIP-constrained local duplication, and ecologically driven adaptive expansion.

Examination of key evolutionary nodes across ecological niches revealed core adaptive strategies underlying *Colletotrichum* diversification. Plants with contrasting growth forms differ in life-history traits, with fast-growing herbaceous plants tending toward quantitative defense compounds and slower-growing woody plants toward qualitative ones (Salguero-Gómez et al. 2016; Givnish 2025). Accordingly, *Colletotrichum* has evolved distinct detoxification strategies: woody host-dominant lineage (WHDL) shows expansion of oxidative enzyme, supporting a steady oxidative modification system targeting aromatic compounds, lignin derivatives, and alkaloids; whereas herbaceous-associated lineages (HAL) preferentially expanded laccases, reductases, and glutathione-dependent enzymes, forming a rapid-response system that efficiently neutralizes reactive phenolics, aldehydes, and ROS. Within HAL, detoxification-related gene contracted widely in monocot-specific but expanded in dicot-specific lineages, particularly nitrilases and aldehyde dehydrogenases, suggesting adaptation to nitrile- and aldehyde-rich dicot defenses.

Host cell wall degradation represents another major axis of adaptation in *Colletotrichum*. WHDL exhibits expansion of GH39 and alkaline protease during diversification, likely enhancing degradation of hemicellulose-rich and lignified cell walls (Vassilev et al. 2012; Dayoub et al. 2024); monocot–dicot differentiation in HAL is characterized by contraction of glucan- and pectin-degrading enzymes in monocots but expansion in dicots, consistent with the lower pectin content in monocot cell wall (Haas and Peaucelle 2021; Zhang et al. 2025). Opposing shifts in RG-I targeting enzymes highlight substrate-specific adaptation, while widespread contraction of cell wall remodeling and others genes in monocot-associated lineages may further constrain host range. By contrast, the broad host-range Glosc lineage exhibited a comprehensive expansion, including oxidative and rapid-response detoxification systems, hemicellulose- and pectin-degrading enzymes, and adhesion proteins, suggesting broad adaptability to diverse host environments. Moreover, contraction of cell wall biosynthesis enzymes (e.g., chitin synthases and 1,3-β-glucan synthases) in the Glosc lineage may reduce immunogenicity (Shree et al. 2024). Across lifestyle transitions, endophytic lineages commonly contracted genes related to cell adhesion, cellulose degradation, and cell wall remodeling, indicating reduced invasiveness and host immune activation (diminished MAMP exposure). Meanwhile, concomitant contraction in core metabolism, transport, and transcriptional regulation may facilitate a stable endophytic state through functional streamlining and improved host compatibility. These patterns underscore how gene family variation shapes ecological diversification in *Colletotrichum*.

## Conclusions

In conclusion, this study supports the revised taxonomic status of *C. parvisporum*, *C. menglaense*, *C. dianense*, *C. dimorphum*, and *C. nanhuaense*, and establishes a comprehensive and robust genome-based phylogenetic framework for the genus *Colletotrichum*. Our analyses reveal substantial interspecific variation in genome size, primarily driven by uncontrolled TE proliferation resulting from RIP inefficiency, shaping genome structural diversity. In addition, *Colletotrichum* exhibits pronounced codon usage bias (CUB) that is largely phylogenetically constrained, tightly linked to strain/species diversification, and showing bistable co-evolutionary with tRNA gene abundance. The secretome and multiple functional gene repertoires (e.g., transporters, transcription factors, and SMBGCs) show coordinated variation in abundance and significant differentiation across species complexes and host types, with phylogenetic shaping most functional category and ecological selection acts on specific modules to drive adaptation. Evolutionary trajectories reveal that widespread gene family variation in *Colletotrichum* underlies diverse host adaptation strategies—host specialization, generalism, and endophytism—accompanied by reconfiguration of detoxification, host invasion, and cell wall-related functions. Collectively, these findings refine the taxonomic relationships within *Colletotrichum* and lay the groundwork for understanding its genome evolution and adaptation strategies.

## Additional information

### Artificial Intelligence (AI) use

The authors accept full responsibility for the content of the manuscript, including the disclosure of any use of AI.

No AI tools were used in the preparation of this manuscript.

### Adherence to national and international regulations

All the fungal strains used in this study have been legally obtained, respecting the Convention on Biological Diversity (Rio Convention).

### Author ORCIDs

J.-X. Shen https://orcid.org/0000-0003-4467-4034

M. Qiao https://orcid.org/0000-0002-7438-1796

X.-W. Dai https://orcid.org/0009-0000-1175-8456

S.-Q. Liu https://orcid.org/0000-0003-2023-4552

Z.-F. Yu https://orcid.org/0000-0002-6246-5861

### Data availability

All of the data that support the findings of this study are available in the main text or Supplementary Information.

## Supplementary materials

Supplementary material 1 Additional figures Data type zip Explanation note **fig. S1**. Phylogenetic tree of *Colletotrichum* calculated with a maximum likelihood analysis of the combined act, chs-1, gapdh, ITS, and tub2 sequence alignment. **fig. S2**. The 150 *Colletotrichum* strains samples were grouped according to four distinct dimensions. **fig. S3**. The correlation between repeat sequence content and gene number. **fig. S4**. Neutral plots of representative *Colletotrichum* strains showing extremes in slope and GC3s span. **fig. S5**. ENc plots of four representative *Colletotrichum* strains showing extremes. **fig. S6**. The relationship between codon usage bias (CUB) and variations in tRNA gene abundance. **fig. S7**. Top 20 codons contributing to PC1 in codon usage principal component analysis (PCA). **fig. S8**. Relationships between the abundance of major categories and total secretome size in *Colletotrichum*. **fig. S9**. Heatmap of functional gene variation across lineages, lifestyles, and hosts in *Colletotrichum*. **fig. S10**. Distances between significantly expanded/contracted genes and nearest repeats with composition in *Colletotrichum*. https://binary.pensoft.net/file/1747467This dataset is made available under the Open Database License (http://opendatacommons.org/licenses/odbl/1.0/). The Open Database License (ODbL) is a license agreement intended to allow users to freely share, modify, and use this Dataset while maintaining this same freedom for others, provided that the original source and author(s) are credited. Jian-Xin Shen, Min Qiao, Jia-Nan Hong, Xin-Wen Dai, Wen-Jie Li, Shu-Qun Liu, Ze-Fen Yu

Supplementary material 2 Identifiers and basic information of *Colletotrichum* Data type pdf Explanation note **table S1**. Identifiers and basic information of Colletotrichum (in-house and database retrieved) https://binary.pensoft.net/file/1747468This dataset is made available under the Open Database License (http://opendatacommons.org/licenses/odbl/1.0/). The Open Database License (ODbL) is a license agreement intended to allow users to freely share, modify, and use this Dataset while maintaining this same freedom for others, provided that the original source and author(s) are credited. Jian-Xin Shen, Min Qiao, Jia-Nan Hong, Xin-Wen Dai, Wen-Jie Li, Shu-Qun Liu, Ze-Fen Yu

Supplementary material 3 Additonal tables Data type xlsx Explanation note **table S2**. Reference species and strains used for homology-based gene prediction in Maker. **table S3**. Functional mapping of transcription factors and effector-related proteins based on Pfam domains. **table S4**. Strain information and GenBank accession numbers of multilocus phylogenetic markers. **table S5**. Taxonomic reassessment of the genome assemblies labelled as C. musae GZ23 and C. liaoningense YN33 based on genome-derived barcode loci. **table S6**. Groups with significant differences in repetitive sequence categories. **table S7**. Blomberg’s K test for 59 codons. **table S8**. Intergroup comparison of codons with weak phylogenetic constraint. **table S9**. Pagel’s λ estimates for 81 major functional categories exhibiting substantial variation across species/strains. **table S10**. Consensus annotations and GO descriptions of significantly expanded and contracted gene families. **table S11**. GO slim categories of significantly expanded and contracted gene families. **table S12**. Significantly expanded gene families (n = 51) at the *Colletotrichum* ancestral node and their consensus annotations. **table S13**. Expanded/contracted gene families linked to host adaptation and cellular processes across *Colletotrichum* lineages https://binary.pensoft.net/file/1747469This dataset is made available under the Open Database License (http://opendatacommons.org/licenses/odbl/1.0/). The Open Database License (ODbL) is a license agreement intended to allow users to freely share, modify, and use this Dataset while maintaining this same freedom for others, provided that the original source and author(s) are credited. Jian-Xin Shen, Min Qiao, Jia-Nan Hong, Xin-Wen Dai, Wen-Jie Li, Shu-Qun Liu, Ze-Fen Yu

Supplementary material 4 Multiple sequence alignments used for phylogenomic analyses Data type fasta https://binary.pensoft.net/file/1747470This dataset is made available under the Open Database License (http://opendatacommons.org/licenses/odbl/1.0/). The Open Database License (ODbL) is a license agreement intended to allow users to freely share, modify, and use this Dataset while maintaining this same freedom for others, provided that the original source and author(s) are credited. Jian-Xin Shen, Min Qiao, Jia-Nan Hong, Xin-Wen Dai, Wen-Jie Li, Shu-Qun Liu, Ze-Fen Yu

Supplementary material 5 Multiple sequence alignments used for phylogenetic analyses Data type fasta https://binary.pensoft.net/file/1747471This dataset is made available under the Open Database License (http://opendatacommons.org/licenses/odbl/1.0/). The Open Database License (ODbL) is a license agreement intended to allow users to freely share, modify, and use this Dataset while maintaining this same freedom for others, provided that the original source and author(s) are credited. Jian-Xin Shen, Min Qiao, Jia-Nan Hong, Xin-Wen Dai, Wen-Jie Li, Shu-Qun Liu, Ze-Fen Yu

Supplementary material 6 Multilocus phylogenetic tree for the taxonomic reassessment of the genome assemblies labelled as *C. musae* GZ23 and *C. liaoningense* YN33 Data type zip https://binary.pensoft.net/file/1747472This dataset is made available under the Open Database License (http://opendatacommons.org/licenses/odbl/1.0/). The Open Database License (ODbL) is a license agreement intended to allow users to freely share, modify, and use this Dataset while maintaining this same freedom for others, provided that the original source and author(s) are credited. Jian-Xin Shen, Min Qiao, Jia-Nan Hong, Xin-Wen Dai, Wen-Jie Li, Shu-Qun Liu, Ze-Fen Yu
