# Supplementary material for: Genus-wide comparative genomics of Colletotrichum reveals evolutionary conservation and divergent ecological adaptations
Source: IMA Fungus. 2026 Aug 17;17:e195303. doi: 10.3897/imafungus.17.195303 (PMC13504312; doi:10.3897/imafungus.17.195303)
Supplement: Supplementary file 1 [file imafungus-17-e195303-s002.pdf]

Supporting file for

Genus-wide comparative genomics of *Colletotrichum* reveals evolutionary conservation and divergent ecological adaptations

Jian-Xin Shen[1,2,3], Min Qiao[1,2], Jia-Nan Hong[1,2,3], Xin-Wen Dai[1,2,3], Wen-Jie Li[1,2,3], Shu-Qun Liu[1,2], and Ze-Fen Yu[1,2]

1 *State Key Laboratory for Conservation and Utilization of Bio-Resources in Yunnan, Yunnan University, Kunming 650504, China*

2 *Yunnan Key Laboratory of Basic Research and Innovative Application for Green Biological Production, Yunnan University, Kunming 650504, China*

3 *School of Life Sciences, Yunnan University, Kunming 650504, China*

Corresponding authors: Shu-Qun Liu (shuqunliu@ynu.edu.cn); Ze-Fen Yu (zfyu2021@163.com)

Table S1. Identifiers and basic information of 150 *Colletotrichum* (in-house and database retrieved)

Species	Strain	Genome size	Complex	Life style	Host	Host life-form	Host clade	NCBI ID	Reference
C. abscissum	IMI 504890	53.94	Acutatum	Pathogen	*Citrus* x *sinensis*	Woody	Dicotyledon	GCF_030869225.1	(Baroncelli et al. 2024)
C. acutatum	CBS 112980	49.06	Acutatum	Pathogen	*Pinus radiata*	Woody	Gymnosperm	GCF_030867785.1	(Baroncelli et al. 2024)
C. acutatum	43380	50.50	Acutatum	Pathogen	*Prunus dulcis*	Woody	Dicotyledon	GCA_020465775.1	(de Silva et al. 2021)
C. acutatum	83	49.75	Acutatum	Pathogen	*Fragaria* x *ananassa*	Herbaceous	Dicotyledon	GCA_021124905.1	(RoyChowdhury et al. 2026)
C. adenophorae	CGMCC 3.18952^{T}	48.44	Undistributed	Endophyte	*Ageratina adenophora*	Herbaceous	Dicotyledon	GCA_058130455.1	In this study
C. aenigma	Cg56	59.19	Gloeosporioides	Pathogen	*Fragaria* x *ananassa*	Herbaceous	Dicotyledon	GCF_013390185.1	(Gan et al. 2021)
C. aeschynomenes	SAR 10-96	56.16	Gloeosporioides	Endophyte	*Hevea brasiliensis*	Woody	Dicotyledon	GCA_025329375.1	(Rehner Stephen et al. 2023)
C. aeschynomenes	SAR 10-98	56.03	Gloeosporioides	Endophyte	*Hevea brasiliensis*	Woody	Dicotyledon	GCA_025329385.1	(Rehner Stephen et al. 2023)
C. agaves	ARM3	71.38	Agaves	Pathogen	*Agave americana*	Woody	Monocotyledon	GCA_036785465.1	PRJNA1007928 (Unpublished)
C. asianum	FJ11	66.12	Gloeosporioides	Pathogen	*Mangifera indica*	Woody	Dicotyledon	GCA_027406305.1	(Wang et al. 2023)
C. asianum	SAR 11-59	65.09	Gloeosporioides	Endophyte	*Mangifera indica*	Woody	Dicotyledon	GCA_025328345.1	(Rehner Stephen et al. 2023)
C. asianum	SAR 11-240	60.04	Gloeosporioides	Endophyte	*Theobroma cacao*	Woody	Dicotyledon	GCA_025328625.1	(Rehner Stephen et al. 2023)
C. australisinense	GX1655	55.3	Acutatum	Pathogen	*Hevea brasiliensis*	Woody	Dicotyledon	GCA_014706365.1	(Liu et al. 2020)
C. baiyuense	CGMCC 3.18941^{T}	50.45	Tibetense	Endophyte	*Hippuris vulgaris*	Herbaceous	Dicotyledon	GCA_058130435.1	In this study
C. brevisporum	C1	62.66	Magnum	Pathogen	*Carica papaya*	Woody	Dicotyledon	GCA_030770045.1	(Yang et al. 2024)
C. cangyuanense	CGMCC 3.18969^{T}	58.38	Gloeosporioides	Endophyte	*Ageratina adenophora*	Herbaceous	Dicotyledon	GCA_056956895.1	In this study
C. camelliae	LS-19	67.47	Gloeosporioides	Pathogen	*Camellia japonica*	Woody	Dicotyledon	GCA_018853505.1	(Leep et al. 2026)
C. casaense	CGMCC 3.18947^{T}	50.3	Tibetense	Endophyte	*Hippuris vulgaris*	Herbaceous	Dicotyledon	GCA_058130395.1	In this study
C. caudatum	CBS 131602^{T}	44.2	Caudatum	Pathogen	*Sorghastrum nutans*	Herbaceous	Monocotyledon	GCA_030867225.1	(Baroncelli et al. 2024)
C. cereale	Tj3	58.52	Graminicola	Unknown	*Avena sativa*	Herbaceous	Monocotyledon	GCA_034509685.1	PRJNA980566 (Unpublished)
C. chlorophyti	NTL11	52.39	Undistributed	Pathogen	*Solanum lycopersicum*	Herbaceous	Dicotyledon	GCA_001937105.1	(Gan et al. 2017)
C. chrysophilum	AFK26	55.97	Gloeosporioides	Pathogen	*Malus domestica*	Woody	Dicotyledon	GCF_026319265.1	(Khodadadi et al. 2023)
C. cliviicola	YN31	52.05	Orchidearum	Pathogen	*Mangifera indica*	Woody	Dicotyledon	GCA_026119605.1	(Wang et al. 2023)
C. coccodes	NJ-RT1	50.12	Undistributed	Pathogen	*Capsicum annuum*	Woody	Dicotyledon	GCA_002249775.1	PRJNA285890 (Unpublished)
C. coccodes	CcND04	50.24	Undistributed	Unknown	*Solanum tuberosum*	Herbaceous	Dicotyledon	GCA_020466075.1	PRJNA755315 (Unpublished)
C. cordylinicola	HN23	61.62	Gloeosporioides	Pathogen	*Mangifera indica*	Woody	Dicotyledon	GCA_027406065.1	(Wang et al. 2023)
C. costaricense	IMI 309622	51.63	Acutatum	Pathogen	*Coffea* sp.	Woody	Dicotyledon	GCF_030867565.1	(Baroncelli et al. 2024)
C. cuscutae	IMI 304802^{T}	80.45	Acutatum	Pathogen	*Cuscuta* sp.	Herbaceous	Dicotyledon	GCA_030869315.1	(Baroncelli et al. 2024)
C. demersi	CGMCC 3.18946^{T}	53.65	Boninense	Endophyte	*Ceratophyllum demersum*	Herbaceous	Monocotyledon	GCA_056956775.1	In this study
C. destructivum	CBS 520.97	51.75	Destructivum	Pathogen	*Medicago sativa*	Herbaceous	Dicotyledon	GCF_034447905.1	(Lapalu et al. 2024)
C. dianense	CGMCC 3.18943^{T}	52	Undistributed	Endophyte	*Alternanthera philoxeroides*	Herbaceous	Dicotyledon	GCA_058130415.1	In this study
C. dimorphum	CGMCC 3.16083^{T}	61.37	Gloeosporioides	Pathogen	*Ageratina adenophora*	Herbaceous	Dicotyledon	GCA_058130375.1	In this study
C. echinochloae	B-48	62.25	Graminicola	Pathogen	*Echinochloa crus-galli*	Herbaceous	Monocotyledon	GCA_016618095.1	(Gu et al. 2024)
C. eleusines	NJC-16	53.51	Graminicola	Pathogen	*Eleusine indica*	Herbaceous	Monocotyledon	GCA_016807845.1	(Gu et al. 2024)
C. endophyticum	YN32	61.44	Gloeosporioides	Pathogen	*Mangifera indica*	Woody	Dicotyledon	GCA_027406105.1	(Wang et al. 2023)
C. eremochloae	CBS 129661^{T}	47.21	Graminicola	Pathogen	*Eremochloa ophiuroides*	Herbaceous	Monocotyledon	GCA_030867245.1	(Baroncelli et al. 2024)
C. euphorbiae	CBS 134725^{T}	66.28	Agaves	Unknown	*Euphorbia* sp.	Unknown	Dicotyledon	GCA_037178605.1	PRJNA1043725 (Unpublished)
C. falcatum	CF08	56.08	Graminicola	Pathogen	*Saccharum officinarum*	Herbaceous	Monocotyledon	GCA_019425465.1	(Chandra et al. 2021)
C. falcatum	Cf671	48.19	Graminicola	Pathogen	*Saccharum officinarum*	Herbaceous	Monocotyledon	GCA_001484525.1	(Chandra et al. 2021)
C. filicis	CBS 101611^{T}	62.97	Acutatum	Pathogen	Fern	Unknown	Tracheophyte	GCA_023376865.1	(Goulin et al. 2022)
C. fioriniae	ACFK16	49.3	Acutatum	Pathogen	*Malus domestica*	Woody	Dicotyledon	GCF_026319165.1	(Khodadadi et al. 2023)
C. fioriniae	IMI 355084	49.45	Acutatum	Endophyte	*Quercus ilex*	Woody	Dicotyledon	GCA_027942845.1	(Hill et al. 2023)
C. fioriniae	PJ7	49	Acutatum	Pathogen	*Fragaria* x *ananassa*	Herbaceous	Dicotyledon	GCA_000582985.1	(Baroncelli et al. 2014)
C. fructicola	CGMCC 3.17371	58.06	Gloeosporioides	Pathogen	*Fragaria* x *ananassa*	Herbaceous	Dicotyledon	GCF_009771025.1	(Armitage Andrew et al. 2020)
C. fructicola	1104-7	58.69	Gloeosporioides	Pahtogen	*Malus domestica*	Woody	Dicotyledon	GCA_002314275.2	(Liang et al. 2020)
C. fructicola	Cf413	56.53	Gloeosporioides	Endophyte	*Fragaria* x *ananassa*	Herbaceous	Dicotyledon	GCA_013390205.1	(Gan et al. 2021)
C. fructicola	Cf415	56.01	Gloeosporioides	Endophyte	*Fragaria* x *ananassa*	Herbaceous	Dicotyledon	GCA_013201905.1	(Gan et al. 2021)
C. fructicola	Cf245	56.06	Gloeosporioides	Endophyte	*Fragaria* x *ananassa*	Herbaceous	Dicotyledon	GCA_013201925.1	(Gan et al. 2021)
C. fructicola	SAR 11-57	55.45	Gloeosporioides	Endophyte	*Persea americana*	Woody	Dicotyledon	GCA_025328355.1	(Rehner Stephen et al. 2023)
C. garzense	CGMCC 3.18948^{T}	49.89	Tibetense	Endophyte	*Hippuris vulgaris*	Herbaceous	Dicotyledon	GCA_058130335.1	In this study
C. gigasporum	JS-0367	87	Gigasporum	Endophyte	*Morus alba*	Woody	Dicotyledon	GCA_024584535.1	PRJNA742180 (Unpublished)
C. gigasporum	HN42	57.89	Gigasporum	Pathogen	*Mangifera indica*	Woody	Dicotyledon	GCA_027406085.1	(Wang et al. 2023)
C. gloeosporioides	Lc1	61.9	Gloeosporioides	Pathogen	*Liriodendron chinense*	Woody	Dicotyledon	GCF_011800055.1	(Fu et al. 2020)
C. gloeosporioides	Cg01	55.77	Gloeosporioides	Endophyte	*Huperzia serrata*	Herbaceous	Tracheophyte	GCA_003666125.1	(Kang et al. 2019)
C. gloeosporioides	CgDa01	62.78	Gloeosporioides	Pathogen	*Dioscorea alata*	Herbaceous	Monocotyledon	GCA_021650765.1	(Wang et al. 2022)
C. gloeosporioides	ES026	58.52	Gloeosporioides	Endophyte	*Huperzia serrata*	Herbaceous	Tracheophyte	GCA_003568745.1	(Xia et al. 2022)
C. gloeosporioides	KY332	58	Gloeosporioides	Pathogen	*Fragaria* x *ananassa*	Herbaceous	Dicotyledon	GCA_014705165.1	(Eaton et al. 2021)
C. gloeosporioides	SMCG1#C	61.92	Gloeosporioides	Pathogen	*Cunninghamia lanceolata*	Woody	Gymnosperm	GCA_003243855.1	(Huang et al. 2019)
C. godetiae	CBS 193.32	51.65	Acutatum	Unknown	Unknown	Unknown	Unknown	GCF_030913485.1	PRJNA333328 (Unpublished)
C. gracile	CGMCC 3.16075^{T}	56.41	Gloeosporioides	Pathogen	*Ageratina adenophora*	Herbaceous	Dicotyledon	GCA_058130355.1	In this study
C. graminicola	M1.001^{T}	51.6	Graminicola	Pathogen	*Zea mays*	Herbaceous	Monocotyledon	GCF_000149035.1	(O'Connell et al. 2012)
C. graminicola	CBS 252.59	44.93	Graminicola	Pathogen	*Zea mays*	Herbaceous	Monocotyledon	GCA_026652245.1	(Rogério et al. 2022)
C. graminicola	T1-3-3	60.7	Graminicola	Pathogen	*Zea mays*	Herbaceous	Monocotyledon	GCA_030463195.1	(Ma et al. 2023)
C. higginsianum	IMI 349063	50.72	Destructivum	Pathogen	*Brassica campestris*	Herbaceous	Dicotyledon	GCF_001672515.1	(O'Connell et al. 2012)
C. horii	PLG3	68.46	Gloeosporioides	Pathogen	*Diospyros* sp.	Woody	Dicotyledon	GCA_027406005.1	(Wang et al. 2023)
C. incanum	MAFF 238704	53.6	Spaethianum	Pathogen	*Raphanus sativus*	Herbaceous	Dicotyledon	GCA_001625285.1	(Hacquard et al. 2016)
C. kahawae	CIFC-Que2	59.07	Gloeosporioides	Pathogen	*Coffea arabica*	Woody	Dicotyledon	GCA_032988895.1	(Pires et al. 2016)
C. karsti	CkLH20	51.85	Boninense	Unknown	*Camellia oleifera*	Woody	Dicotyledon	GCF_011947395.1	PRJNA613862 (Unpublished)
C. ledebouriae	CBS 141284^{T}	146	Agaves	Pathogen	*Ledebouria floribunda*	Herbaceous	Monocotyledon	GCA_036785755.1	PRJNA1008866 (Unpublished)
C. lentis	CT-30	56.1	Destructivum	Pathogen	*Lens culinaris*	Herbaceous	Dicotyledon	GCA_003386485.1	(Bhadauria et al. 2019)
C. liaoningense	YN33	51.85	Acutatum	Pathogen	*Mangifera indica*	Woody	Dicotyledon	GCA_027406045.1	(Wang et al. 2023)
C. limetticola	KLA-Anderson	50.48	Acutatum	Pathogen	*Citrus* x *aurantiifolia*	Woody	Dicotyledon	GCA_030413865.1	(Menicucci et al. 2023)
C. lindemuthianum	89 A2 2-3	99.17	Orbiculare	Pathogen	*Phaseolus vulgaris*	Herbaceous	Dicotyledon	GCA_001693025.2	(de Queiroz Casley et al. 2017)
C. lini	757	54.02	Destructivum	Pathogen	*Linum usitatissimum*	Herbaceous	Dicotyledon	GCA_034638275.1	(Dvorianinova et al. 2024)
C. liriopes	MAFF 242679	52.97	Spaethianum	Pathogen	*Rohdea japonica*	Herbaceous	Monocotyledon	GCA_022179045.1	(Hiruma et al. 2023)
C. lupini	IMI 504893	63.41	Acutatum	Pathogen	*Lupinus albus*	Herbaceous	Dicotyledon	GCF_023278565.1	(Baroncelli et al. 2021)
C. magnum	WT	57.11	Magnum	Unknown	*Citrullus lanatus*	Herbaceous	Dicotyledon	GCA_022457145.1	PRJNA777582 (Unpublished)
C. melonis	Col 31	50.14	Acutatum	Unknown	*Malus domestica*	Woody	Dicotyledon	GCA_030868995.1	(Baroncelli et al. 2024)
C. menglaense	CGMCC 3.18958^{T}	59.29	Gloeosporioides	Unknown	Unknown	Unknown	Unknown	GCA_058130315.1	In this study
C. musae	GZ23	60.48	Magnum	Pathogen	*Mangifera indica*	Woody	Dicotyledon	GCA_027406025.1	(Wang et al. 2023)
C. musae	GM20	49.12	Gloeosporioides	Pathogen	*Musa* spp.	Herbaceous	Monocotyledon	GCA_002814275.1	(Silva Junior et al. 2018)
C. musicola	LFN0074	52.73	Orchidearum	Pathogen	*Glycine max*	Herbaceous	Dicotyledon	GCA_014235935.1	(Rogério et al. 2020)
C. nanhuaense	CGMCC 3.18962^{T}	60.09	Gloeosporioides	Pathogen	*Ageratina adenophora*	Herbaceous	Dicotyledon	GCA_056956795.1	In this study
C. navitas	CBS 125086^{T}	52.43	Graminicola	Pathogen	*Panicum virgatum*	Herbaceous	Monocotyledon	GCF_030913465.1	(Baroncelli et al. 2024)
C. neosansevieriae	CBS 139918^{T}	78.94	Agaves	Pathogen	*Sansevieria trifasciata*	Herbaceous	Monocotyledon	GCA_036785495.1	PRJNA1009234 (Unpublished)
C. noveboracense	PMBrms-1	59.09	Gloeosporioides	Pathogen	*Malus domestica*	Woody	Dicotyledon	GCA_026319125.1	(Khodadadi et al. 2023)
C. nupharicola	CBS 470^{T}	58.76	Gloeosporioides	Pathogen	*Nuphar lutea*	Herbaceous	Dicotyledon	GCA_026319135.1	(Khodadadi et al. 2023)
C. nupharicola	Coll922	72.74	Gloeosporioides	Pathogen	*Nuphar lutea*	Herbaceous	Dicotyledon	GCA_026319225.1	(Khodadadi et al. 2023)
C. nymphaeae	HC646	51.26	Acutatum	Pathogen	*Malus domestica*	Woody	Dicotyledon	GCA_014705455.1	(Eaton et al. 2021)
C. nymphaeae	JS-0361	50.07	Acutatum	Unknown	*Morus alba*	Woody	Dicotyledon	GCA_029169025.1	PRJNA784994 (Unpublished)
C. nymphaeae	KY613	50.58	Acutatum	Pathogen	*Fragaria* x *ananassa*	Herbaceous	Dicotyledon	GCA_014705085.1	(Eaton et al. 2021)
C. oblongisporum	CGMCC 3.16074^{T}	57.27	Gloeosporioides	Pathogen	*Ageratina adenophora*	Herbaceous	Dicotyledon	GCA_056956705.1	In this study
C. orbiculare MAFF 240422	104-T	89.75	Orbiculare	Pathogen	*Cucumis sativus*	Herbaceous	Dicotyledon	GCA_000350065.2	(Gan et al. 2013)
C. orchidophilum	IMI 309357	48.56	Undistributed	Unknown	*Phalaenopsis* sp.	Unknown	Monocotyledon	GCF_001831195.1	(Baroncelli et al. 2018)
C. Paranaense	IMI 384185	49.18	Acutatum	Pathogen	*Caryocar brasiliense*	Woody	Dicotyledon	GCF_030867605.1	(Baroncelli et al. 2024)

C. parvisporum	CGMCC 3.16078 ^{T}	58.87	Gloeosporioides	Pathogen	*Ageratina adenophora*	Herbaceous	Dicotyledon	GCA_058130295.1	In this study
C. philoxeroidis	CGMCC 3.18945 ^{T}	51.89	Magnum	Endophyte	*Alternanthera philoxeroides*	Herbaceous	Dicotyledon	GCA_058130275.1	In this study
C. phormii	CBS 102054	51.67	Acutatum	Pthogen	*Phormium* spp.	Herbaceous	Monocotyledon	GCF_030913505.1	(Baroncelli et al. 2024)
C. plurivorum	LFN00145	49.7	Orchidearum	Pathogen	*Glycine max*	Herbaceous	Dicotyledon	GCA_014235945.1	(Rogério et al. 2020)
C. salicis	CBS 607.94 ^{T}	48.37	Acutatum	Pathogen	*Salix* spp.	Woody	Dicotyledon	GCA_001563125.1	PRJNA238477 (Unpublished)
C. sansevieriae	FPH2021-6	98.57	Agaves	Pathogen	*Sansevieria trifasciata*	Herbaceous	Monocotyledon	GCA_026260285.1	(David et al. 2022)
C. sansevieriae	Sa-1-2 ^{T}	51.2	Agaves	Pathogen	*Sansevieria trifasciata*	Herbaceous	Monocotyledon	GCA_002749775.1	(Nakamura et al. 2018)
C. scovillei	TJNH1	52	Acutatum	Pathogen	*Capsicum annuum*	Herbaceous	Dicotyledon	GCF_011075155.1	(Huo et al. 2020)
C. scovillei	YN51	52.2	Acutatum	Pathogen	*Mangifera indica*	Woody	Dicotyledon	GCA_026540245.1	(Wang et al. 2023)
C. shisoi	PG-2018a	69.67	Destructivum	Pathogen	*Perilla frutescens* var. *crispa*	Herbaceous	Dicotyledon	GCA_006783085.1	(Gan et al. 2019a)
C. siamense	Cg363	62.94	Gloeosporioides	Pathogen	*Fragaria* x *ananassa*	Herbaceous	Dicotyledon	GCF_013390195.1	(Gan et al. 2021)
C. siamense	ICMP 18578 ^{T}	55.96	Gloeosporioides	Pathogen	*Coffea arabica* L.	Woody	Dicotyledon	GCA_008520285.1	(Meng et al. 2019)
C. siamense	SAR 10-65	59.66	Gloeosporioides	Endophyte	*Hevea brasiliensis*	Woody	Dicotyledon	GCA_025328335.1	(Rehner Stephen et al. 2023)
C. siamense	SAR 10-70	59.54	Gloeosporioides	Endophyte	*Hevea brasiliensis*	Woody	Dicotyledon	GCA_025327165.1	(Rehner Stephen et al. 2023)
C. siamense	SAR 10-71	59.86	Gloeosporioides	Endophyte	*Hevea brasiliensis*	Woody	Dicotyledon	GCA_025327135.1	(Rehner Stephen et al. 2023)
C. siamense	SAR 10-75	60.81	Gloeosporioides	Endophyte	*Hevea brasiliensis*	Woody	Dicotyledon	GCA_025327155.1	(Rehner Stephen et al. 2023)
C. siamense	SAR 10-76	60.13	Gloeosporioides	Endophyte	*Hevea brasiliensis*	Woody	Dicotyledon	GCA_025327145.1	(Rehner Stephen et al. 2023)
C. siamense	SAR 10-77	60.54	Gloeosporioides	Endophyte	*Hevea brasiliensis*	Woody	Dicotyledon	GCA_025330015.1	(Rehner Stephen et al. 2023)
C. siamense	SAR 10-86	60.72	Gloeosporioides	Endophyte	*Hevea brasiliensis*	Woody	Dicotyledon	GCA_025327125.1	(Rehner Stephen et al. 2023)
C. siamense	SAR 11-239	58.1	Gloeosporioides	Endophyte	*Theobroma cacao*	Woody	Dicotyledon	GCA_025328985.1	(Rehner Stephen et al. 2023)
C. sidae	CBS 518.97	86.83	Orbiculare	Unknown	*Sida spinosa*	Herbaceous	Dicotyledon	GCA_004367935.1	(Gan et al. 2019b)
C. simmondsii	CBS 122122 ^{T}	50.47	Acutatum	Pathogen	*Carica papaya*	Woody	Dicotyledon	GCA_001563135.1	PRJNA239224 (Unpublished)
C. sojae	LFN0009	49.35	Orchidearum	Pathogen	*Glycine max*	Herbaceous	Dicotyledon	GCA_014235955.1	(Rogério et al. 2020)
C. somersetense	CBS 131599 ^{T}	53.67	Caudatum	Pathogen	*Sorghastrum nutans*	Herbaceous	Monocotyledon	GCA_030867255.1	(Baroncelli et al. 2024)
Colletotrichum sp.	COLG25	56.92	Gloeosporioides	Pathogen	*Citrus* spp.	Woody	Dicotyledon	GCA_009801095.1	PRJNA554995 (Unpublished)
Colletotrichum sp.	JS-367	87.2	Gigasporum	Endophyte	*Morus* spp.	Woody	Dicotyledon	GCA_003122705.1	PRJNA368787 (Unpublished)
Colletotrichum sp.	SAR 10-66	61.04	Gloeosporioides	Unknown	*Hevea brasiliensis*	Woody	Dicotyledon	GCA_028639655.1	PRJNA728692 (Unpublished)
Colletotrichum sp.	SAR 10-99	56.74	Gloeosporioides	Unknown	*Hevea brasiliensis*	Woody	Dicotyledon	GCA_028654355.1	PRJNA728692 (Unpublished)
C. spaethianum	MAFF 239500	50.92	Spaethianum	Pathogen	*Polygonatum falcatum*	Herbaceous	Monocotyledon	GCF_022836535.1	(Utami and Hiruma 2022)
C. speciosum	CGMCC 3.16081 ^{T}	49.67	Acutatum	Endophyte	*Ageratina adenophora*	Herbaceous	Dicotyledon	GCA_058130255.1	In this study
C. spicati	CGMCC 3.18942 ^{T}	53.08	Boninense	Endophyte	*Myriophyllum spicatum*	Herbaceous	Dicotyledon	GCA_058130235.1	In this study
C. spinosum	CBS 525.97	82.73	Orbiculare	Pathogen	*Xanthium spinosum*	Herbaceous	Dicotyledon	GCA_004366825.1	(Gan et al. 2019b)
C. subhenanense	CGMCC 3.16073 ^{T}	58.87	Gloeosporioides	Pathogen	*Ageratina adenophora*	Herbaceous	Dicotyledon	GCA_058130215.1	In this study
C. sublineola	CsGL1	67.67	Graminicola	Pathogen	*Sorghum bicolor*	Herbaceous	Monocotyledon	GCA_020631755.1	(Baldrich et al. 2021)
C. sublineola	S3.001 ^{T}	46.92	Graminicola	Pathogen	*Sorghum bicolor*	Herbaceous	Monocotyledon	GCA_030867325.1	(Baroncelli et al. 2024)
C. tabacum	YC1	52.15	Destructivum	Pathogen	*Nicotiana tabacum*	Herbaceous	Dicotyledon	GCA_036851105.1	PRJNA962315 (Unpublished)
C. tamarilloi	Tom-12	52.07	Acutatum	Pathogen	*Solanum betaceum*	Woody	Dicotyledon	GCF_030869305.1	PRJNA348678 (Unpublished)
C. tanaceti	BRIP57314 ^{T}	57.91	Destructivum	Pathogen	*Tanacetum cinerariifolium*	Herbaceous	Dicotyledon	GCA_005350895.1	(Lelwala et al. 2019)
C. tengchongense	CGMCC 3.18950 ^{T}	56.97	Gloeosporioides	Endophyte	*Isoetes sinensis*	Herbaceous	Tracheophyte	GCA_058130175.1	In this study
C. theobromicola	CF75	55.88	Gloeosporioides	Pathogen	*Fragaria* x *ananassa*	Herbaceous	Dicotyledon	GCA_014705045.1	(Eaton et al. 2021)
C. theobromicola	KY152	56.53	Gloeosporioides	Pathogen	*Malus domestica*	Woody	Dicotyledon	GCA_014705415.1	(Eaton et al. 2021)
C. tofieldiae	0861	52.99	Spaethianum	Endophyte	*Arabidopsis thaliana*	Herbaceous	Dicotyledon	GCA_022836595.1	(Hiruma et al. 2023)
C. tofieldiae	0861*	52.84	Spaethianum	Endophyte	*Arabidopsis thaliana*	Herbaceous	Dicotyledon	GCA_001625265.1	(Hacquard et al. 2016)
C. tofieldiae	CBS 127615	52.72	Spaethianum	Unknown	*Agapanthus* sp.	Herbaceous	Monocotyledon	GCA_001618715.1	(Hacquard et al. 2016)
C. tofieldiae	CBS 168.49	52.96	Spaethianum	Unknown	*Lupinus polyphyllus*	Herbaceous	Dicotyledon	GCA_001618705.1	(Hacquard et al. 2016)
C. tofieldiae	MAFF 712333	54.25	Spaethianum	Pathogen	*Eliokarmos dubius*	Herbaceous	Monocotyledon	GCA_022836555.1	(Hiruma et al. 2023)
C. trifolii	543-2	109.7	Orbiculare	Pathogen	*Medicago sativa*	Herbaceous	Dicotyledon	GCA_004367215.1	(Gan et al. 2019b)
C. tropicale	HN32	56.94	Gloeosporioides	Pathogen	*Mangifera indica*	Woody	Dicotyledon	GCA_027406125.1	(Wang et al. 2023)
C. tropicicola	FJ4	55.11	Dracaenophilum	Unknown	*Guanxi pomelo*	Woody	Monocotyledon	GCA_025962755.1	PRJNA889704 (Unpublished)
C. truncatum	CMES 1059	56.1	Truncatum	Pathogen	*Glycine max*	Herbaceous	Dicotyledon	GCF_014235925.1	(Rogério et al. 2020)
C. truncatum	KLC.C-4	44.15	Truncatum	Unknown	*Capsicum annuum*	Herbaceous	Dicotyledon	GCA_011947275.1	PRJNA594831 (Unpublished)
C. viniferum	CvYL2a	73.41	Gloeosporioides	Pathogen	*Vitis vinifera* cv. *Kyoho*	Woody	Dicotyledon	GCA_020226115.1	(Dou et al. 2021)
C. vulgaris	CGMCC 3.18940 ^{T}	49.17	Tibetense	Endophyte	*Hippuris vulgaris*	Herbaceous	Dicotyledon	GCA_058130195.1	In this study
C. yuanjiangense	CGMCC 3.18964 ^{T}	57.72	Gloeosporioides	Pathogen	*Ageratina adenophora*	Herbaceous	Dicotyledon	GCA_058130155.1	In this study
C. zoysiae	MAFF 238573 ^{T}	46.53	Caudatum	Pathogen	*Zoysia tenuifolia*	Herbaceous	Monocotyledon	GCA_030867305.1	(Baroncelli et al. 2024)

References

Armitage Andrew D, Nellist Charlotte F, Bates Helen J, et al. (2020) Draft genome sequence of the strawberry anthracnose pathogen *Colletotrichum fructicola*. Microbiology Resource Announcements 9 (12):10.1128/mra.01598-01519. https://doi.org/10.1128/mra.01598-19

Baldrich P, Chaya T, Caplan JL, et al. (2021) Genome assembly and transcriptome of *Colletotrichum sublineola* CsGL1, a new resource to study anthracnose disease in sorghum. Molecular Plant-Microbe Interactions 34 (10):1209–1211. https://doi.org/10.1094/MPMI-04-21-0094-A

Baroncelli R, Cobo-Díaz JF, Benocci T, et al. (2024) Genome evolution and transcriptome plasticity is associated with adaptation to monocot and dicot plants in *Colletotrichum* fungi. Gigascience 13:giae036. https://doi.org/10.1093/gigascience/giae036

Baroncelli R, Pensec F, Da Lio D, et al. (2021) Complete genome sequence of the plant-

pathogenic fungus *Colletotrichum lupini*. Molecular Plant-Microbe Interactions 34 (12):1461–1464. https://doi.org/10.1094/MPMI-07-21-0173-A

Baroncelli R, Sreenivasaprasad S, Sukno Serenella A, et al. (2014) Draft genome sequence of *Colletotrichum acutatum* Sensu Lato (*Colletotrichum fioriniae*). Genome Announcements 2 (2):10.1128/genomea.00112-00114. https://doi.org/10.1128/genomea.00112-14

Baroncelli R, Sukno SA, Sarrocco S, et al. (2018) Whole-genome sequence of the orchid anthracnose pathogen *Colletotrichum orchidophilum*. Molecular Plant-Microbe Interactions 31 (10):979–981. https://doi.org/10.1094/MPMI-03-18-0055-A

Bhadauria V, MacLachlan R, Pozniak C, et al. (2019) Genetic map-guided genome assembly reveals a virulence-governing minichromosome in the lentil anthracnose pathogen *Colletotrichum lentis*. New Phytologist 221:431–445. https://doi.org/10.1111/nph.15369

Chandra A, Singh D, Joshi D, et al. (2021) A highly contiguous reference genome assembly for *Colletotrichum falcatum* pathotype Cf08 causing red rot disease in sugarcane. 3 Biotech 11 (3):148. https://doi.org/10.1007/s13205-021-02695-x

David GEV, Emanuel IB, Martin DE, et al. (2022) First report of *Colletotrichum sansevieriae* causing anthracnose of snake plant (*Dracaena trifasciata*) in ohio and its draft genome. Plant Disease 107 (7):2252. https://doi.org/10.1094/PDIS-10-22-2476-PDN

de Queiroz Casley B, Correia Hilberty LN, Menicucci Renato P, et al. (2017) Draft genome sequences of two isolates of *Colletotrichum lindemuthianum*, the causal agent of anthracnose in common beans. Genome Announcements 5 (18):10.1128/genomea.00214-00217. https://doi.org/10.1128/genomea.00214-17

de Silva DD, Mann RC, Kaur J, et al. (2021) Revisiting the *Colletotrichum* species causing anthracnose of almond in Australia. Australasian Plant Pathology 50 (3):267–279. https://doi.org/10.1007/s13313-020-00765-x

Dou MR, Hao Y, Yang J, et al. (2021) Genome sequence resource for *Colletotrichum viniferum*, the cause of grapevine ripe rot in China. Molecular Plant-Microbe Interactions 35 (1):90–93. https://doi.org/10.1094/MPMI-04-21-0077-A

Dvorianinova EM, Sigova EA, Mollaev TD, et al. (2024) Comparative genomic analysis of *Colletotrichum lini* strains with different virulence on flax. Journal of Fungi 10 (1):32. https://doi.org/10.3390/jof10010032

Eaton MJ, Edwards S, Inocencio HA, et al. (2021) Diversity and cross-infection potential of *Colletotrichum* causing fruit rots in mixed-fruit orchards in Kentucky. Plant Disease 105 (4):1115–1128. https://doi.org/10.1094/PDIS-06-20-1273-RE

Fu FF, Hao ZD, Wang PK, et al. (2020) Genome sequence and comparative analysis of *Colletotrichum gloeosporioides* isolated from *Liriodendron* leaves. Phytopathology 110 (7):1260–1269. https://doi.org/10.1094/PHYTO-12-19-0452-R

Gan P, Hiroyama R, Tsushima A, et al. (2021) Telomeres and a repeat-rich chromosome encode effector gene clusters in plant pathogenic *Colletotrichum* fungi. Environmental Microbiology 23 (10):6004–6018. https://doi.org/10.1111/1462-2920.15490

Gan P, Ikeda K, Irieda H, et al. (2013) Comparative genomic and transcriptomic analyses reveal the hemibiotrophic stage shift of *Colletotrichum* fungi. New Phytologist 197 (4):1236–1249. https://doi.org/10.1111/nph.12085

Gan P, Narusaka M, Tsushima A, et al. (2017) Draft genome assembly of *Colletotrichum chlorophyti*, a pathogen of herbaceous plants. Genome Announcements 5 (10):10.1128/genomea.01733-01716. https://doi.org/10.1128/genomea.01733-16

Gan P, Tsushima A, Hiroyama R, et al. (2019a) *Colletotrichum shisoi* sp. nov., an anthracnose pathogen of *Perilla frutescens* in Japan: Molecular phylogenetic, morphological and genomic evidence. Scientific Reports 9:13349. https://doi.org/10.1038/s41598-019-50076-5

Gan P, Tsushima A, Narusaka M, et al. (2019b) Genome sequence resources for four phytopathogenic fungi from the *Colletotrichum orbiculare* species complex. Molecular Plant-Microbe Interactions 32 (9):1088–1090. https://doi.org/10.1094/MPMI-12-18-0352-A

Goulin E, Boufleur TR, Negrini F, et al. (2022) Genome sequence resources of *Colletotrichum abscissum*, the causal agent of citrus post-bloom fruit drop, and the closely related species *C. filicis*. Phytopathology 113 (1):104–107. https://doi.org/10.1094/PHYTO-05-22-0176-A

Gu QN, Chu SH, Huang QC, et al. (2024) Genome sequences of *Colletotrichum eleusines* and *C. echinochloae*: Two pathogens from Poaceae weeds. Microbiology Resource Announcements 13 (3):e00722-23. https://doi.org/10.1128/MRA.00722-23

Hacquard S, Kracher B, Hiruma K, et al. (2016) Survival trade-offs in plant roots during colonization by closely related beneficial and pathogenic fungi. Nature Communications 7:11362. https://doi.org/10.1038/ncomms11362

Hill R, Levicky Q, Pitsillides F, et al. (2023) Tapping culture collections for fungal endophytes: First genome assemblies for three genera and five species in the Ascomycota. Genome Biology and Evolution 15 (3):evad038. https://doi.org/10.1093/gbe/evad038

Hiruma K, Aoki S, Takino J, et al. (2023) A fungal sesquiterpene biosynthesis gene cluster critical for mutualist-pathogen transition in *Colletotrichum tofieldiae*. Nature Communications 14:5288. https://doi.org/10.1038/s41467-023-40867-w

Huang L, Kim KT, Yang JY, et al. (2019) A high-quality draft genome sequence of *Colletotrichum gloeosporioides* sensu stricto SMCG1#C, a causal agent of anthracnose on *Cunninghamia lanceolata* in China. Moleculae Plant-Microbe Interactions 32 (2):139–141. https://doi.org/10.1094/MPMI-05-18-0144-A

Huo JF, Wang YF, Hao YJ, et al. (2020) Genome sequence resource for *Colletotrichum scovillei*, the cause of anthracnose disease of chili. Molecular Plant-Microbe

Interactions 34 (1):122–126. https://doi.org/10.1094/MPMI-03-20-0055-A

Kang XC, Liu CC, Shen PY, et al. (2019) Genomic characterization provides new insights into the biosynthesis of the secondary metabolite Huperzine a in the endophyte *Colletotrichum gloeosporioides* Cg01. Frontiers in Microbiology 9:3237. https://doi.org/10.3389/fmicb.2018.03237

Khodadadi F, Giroux E, Bilodeau GJ, et al. (2023) Genomic resources of four *Colletotrichum* species (*C. fioriniae*, *C. chrysophilum*, *C. noveboracense*, and *C. nupharicola*) threatening commercial apple production in the eastern United States. Molecular Plant-Microbe Interactions 36 (8):529–532. https://doi.org/10.1094/MPMI-10-22-0204-A

Lapalu N, Simon A, Lu A, et al. (2024) Complete genome of the Medicago anthracnose fungus, *Colletotrichum destructivum*, reveals a mini-chromosome-like region within a core chromosome. Microbial Genomics 10 (8):0001283. https://doi.org/10.1099/mgen.0.001283

Leep KR, Arias RS, Copes WE, et al. (2026) Draft genome sequence data of *Colletotrichum camelliae* isolated from *Camellia japonica* in the United States. Data in Brief 66:112613. https://doi.org/10.1016/j.dib.2026.112613

Lelwala RV, Korhonen PK, Young ND, et al. (2019) Comparative genome analysis indicates high evolutionary potential of pathogenicity genes in *Colletotrichum tanaceti*. PLoS One 14 (5):e0212248. https://doi.org/10.1371/journal.pone.0212248

Liang XF, Cao MY, Li S, et al. (2020) Highly contiguous genome resource of *Colletotrichum fructicola* generated using long-read sequencing. Molecular Plant-Microbe Interactions 33 (6):790–793. https://doi.org/10.1094/MPMI-11-19-0316-A

Liu XB, Li BX, Yang Y, et al. (2020) Pathogenic adaptations revealed by comparative genome analyses of two *Colletotrichum* spp., the causal agent of anthracnose in rubber tree. Frontiers in Microbiology 11:1484. https://doi.org/10.3389/fmicb.2020.01484

Ma WD, Yang J, Ding JQ, et al. (2023) CRISPR/Cas9-mediated deletion of large chromosomal segments identifies a minichromosome modulating the *Colletotrichum graminicola* virulence on maize. International Journal of Biological Macromolecules 245:125462. https://doi.org/10.1016/j.ijbiomac.2023.125462

Meng Y, Gleason ML, Zhang R, et al. (2019) Genome sequence resource of the wide-host-range anthracnose pathogen *Colletotrichum siamense*. Molecular Plant-Microbe Interactions 32 (8):931–934. https://doi.org/10.1094/MPMI-01-19-0010-A

Menicucci A, Tikami I, Benocci T, et al. (2023) Draft genome sequence of the keylime (*Citrus × aurantiifolia*) pathogen *Colletotrichum limetticola*. Journal of Plant Pathology 105 (3):1145–1148. https://doi.org/10.1007/s42161-023-01439-5

Nakamura M, Fujikawa T, Nakamori D, et al. (2018) Draft genome sequence of *Colletotrichum sansevieriae* Sa-1–2, the anthracnose pathogen of *Sansevieria trifasciata*. Data in Brief 18:691–695. https://doi.org/10.1016/j.dib.2018.03.083

O'Connell RJ, Thon MR, Hacquard S, et al. (2012) Lifestyle transitions in plant pathogenic *Colletotrichum* fungi deciphered by genome and transcriptome analyses. Nature Genetics 44 (9):1060–1065. https://doi.org/10.1038/ng.2372

Pires AS, Azinheira HG, Cabral A, et al. (2016) Cytogenomic characterization of *Colletotrichum kahawae*, the causal agent of coffee berry disease, reveals diversity in minichromosome profiles and genome size expansion. Plant Pathology 65 (6):968–977. https://doi.org/10.1111/ppa.12479

Rehner Stephen A, Gazis R, Doyle Vinson P, et al. (2023) Genome resources for the *Colletotrichum gloeosporioides* species complex: 13 tree endophytes from the neotropics and paleotropics. Microbiology Resource Announcements 12 (4):e01040-22. https://doi.org/10.1128/mra.01040-22

Rogério F, Baroncelli R, Cuevas-Fernández Francisco B, et al. (2022) Population genomics provide insights into the global genetic structure of *Colletotrichum graminicola*, the causal agent of maize anthracnose. mBio 14 (1):e02878-22. https://doi.org/10.1128/mbio.02878-22

Rogério F, Boufleur TR, Ciampi-Guillardi M, et al. (2020) Genome sequence resources of *Colletotrichum truncatum*, *C. plurivorum*, *C. musicola*, and *C. sojae*: Four species pathogenic to soybean (*Glycine max*). Phytopathology 110 (9):1497–1499. https://doi.org/10.1094/PHYTO-03-20-0102-A

RoyChowdhury M, Sternhagen J, Kim Y (2026) The draft genome sequence of the ascomycete fungus *Colletotrichum acutatum* obtained from strawberry roots. Microbiology Resource Announcements 0:e01357-22. https://doi.org/10.1128/mra.01357-22

Silva Junior WJD, Falcão RM, Sousa-Paula LC, et al. (2018) Draft genome assembly of *Colletotrichum musae*, the pathogen of banana fruit. Data in Brief 17:256–260. https://doi.org/10.1016/j.dib.2018.01.002

Utami YD, Hiruma K (2022) Genome resource of *Colletotrichum spaethianum*, the causal agent of leaf anthracnose in *Polygonatum falcatum*. PhytoFrontiers 2 (2):152–155. https://doi.org/10.1094/PHYTOFR-12-21-0082-A

Wang HM, Huang R, Ren JY, et al. (2023) The evolution of mini-chromosomes in the fungal genus *Colletotrichum*. mBio 14 (4):e00629-23. https://doi.org/10.1128/mbio.00629-23

Wang Y, Xu WT, Lu RS, et al. (2022) Genome sequence resource for *Colletotrichum gloeosporioides*, an important pathogenic fungus causing anthracnose of *Dioscorea alata*. Plant Disease 107 (3):893–895. https://doi.org/10.1094/PDIS-03-22-0567-A

Xia HY, Noushahi HA, Khan AH, et al. (2022) Genome sequencing of *Colletotrichum gloeosporioides* ES026 reveals plausible pathway of HupA. Molecular Biology Reports 49 (12):11611–11622. https://doi.org/10.1007/s11033-022-07850-y

Yang M, Zhou CP, Kuang RB, et al. (2024) Whole-genome sequence of *Colletotrichum brevisporum* causing anthracnose on papaya (*Carica papaya*). PhytoFrontiers 4 (2):236–238. https://doi.org/10.1094/PHYTOFR-07-23-0080-A
